# Development of Novel 1,3-Disubstituted-2-Thiohydantoin Analogues with Potent Anti-Inflammatory Activity; *In Vitro* and *In Silico* Assessments

**DOI:** 10.3390/molecules27196271

**Published:** 2022-09-23

**Authors:** Salma M. Khirallah, Heba M. M. Ramadan, Ahmed Shawky, Safa H. Qahl, Roua S. Baty, Nada Alqadri, Amnah Mohammed Alsuhaibani, Mariusz Jaremko, Abdul-Hamid Emwas, Essa M. Saied

**Affiliations:** 1 Biochemistry Division, Chemistry Department, Faculty of Science, Port Said University, Port Said 42526, Egypt; 2 Chemistry Department, Faculty of Science, Port Said University, Port Said 42526, Egypt; 3 Department of Dermatology, Faculty of Medicine, Al-Azhar University, Cairo 11651, Egypt; 4 Department of Biology, College of Science, University of Jeddah, P.O. Box 80327, Jeddah 21589, Saudi Arabia; 5 Department of Biotechnology, College of Science, Taif University, P.O. Box 11099, Taif 21944, Saudi Arabia; 6 Department of Biology, Turabah University College, Taif University, P.O. Box 11099, Taif 21944, Saudi Arabia; 7 Department of Physical Sport Science, College of Education, Princess Nourah Bint Abdulrahman University, P.O. Box 84428, Riyadh 11671, Saudi Arabia; 8 Smart-Health Initiative and Red Sea Research Center, Division of Biological and Environmental Sciences and Engineering, King Abdullah University of Science and Technology, P.O. Box 4700, Thuwal 23955-6900, Saudi Arabia; 9 Advanced Nanofabrication Imaging and Characterization Center, King Abdullah University of Science and Technology, Core Labs, Thuwal 23955-6900, Saudi Arabia; 10 Chemistry Department, Faculty of Science, Suez Canal University, Ismailia 41522, Egypt; 11 Institute for Chemistry, Humboldt Universität zu Berlin, Brook-Taylor-Str. 2, 12489 Berlin, Germany

**Keywords:** inflammation, NSAID, 2-thiohydantoin, cytotoxicity, inflammatory cytokines, COX enzymes, molecular docking

## Abstract

Inflammation is the main cause of several autoimmune diseases, including type I diabetes, rheumatoid arthritis, bullous pemphigoid, paraneoplastic pemphigoid, and multiple sclerosis. Currently, there is an urgent demand for the discovery of novel anti-inflammatory drugs with potent activity but also safe for long-term application. Toward this aim, the present study reported the design, synthesis, and characterization of a set of novel 1,3-disubstituted-2-thiohydantoins derivatives. The anti-inflammatory activity of synthesized compounds was assessed against murine leukemia cell line (RAW264.7) by evaluating the cytotoxicity activity and their potency to prevent nitric oxide (NO) production. The results revealed that the synthesized compounds possess a considerable cytotoxic activity together with the ability to reduce the NO production in murine leukemia cell line (RAW264.7). Among synthesized compounds, compound **7** exhibited the most potent cytotoxic activity with IC_50_ of 197.68 μg/mL, compared to celecoxib drug (IC_50_ value 251.2 μg/mL), and demonstrated a significant ability to diminish the NO production (six-fold reduction). Exploring the mode of action responsible for the anti-inflammatory activity revealed that compound **7** displays a significant and dose-dependent inhibitory effect on the expression of pro-inflammatory cytokines IL-1β. Furthermore, compound **7** demonstrated the ability to significantly reduce the expression of the inflammatory cytokines IL-6 and TNF-α at 50 μg/mL, as compared to Celecoxib. Finally, detailed molecular modelling studies indicated that compound **7** exhibits a substantial binding affinity toward the binding pocket of the cyclooxygenase 2 enzyme. Taken together, our study reveals that 1,3-disubstituted-2-thiohydantoin could be considered as a promising scaffold for the development of potent anti-inflammatory agents.

## 1. Introduction

Inflammation is a necessary immunological reaction to tissue damage and microbial infections [1]. Several autoimmune diseases including type I diabetes, rheumatoid arthritis, and multiple sclerosis can develop as a result of inflammation. The most likely connection between type 2 diabetes and rheumatoid arthritis involves inflammation and the accumulation of cytokines such as tumor necrosis factor (TNF). The association between rheumatoid arthritis and systemic lupus erythematosus has been reported, as well as between Type 1 diabetes mellitus and multiple sclerosis [2]. One of the most essential aspects of the inflammatory process is the production of prostaglandins, which are produced inside the cells of the body by the cyclooxygenase (Cox) enzyme. Prostaglandins, which cause fever, pain and inflammation, are produced by both the Cox-1 (constitutive) and Cox-2 (stimulated by various pro-inflammatory factors) enzymes [3]. At the inflammatory location, the activated leucocytes create cytokines that improve inflammatory response, causing infiltration of lymphocytes, enzymatic degradation, oedema, and alteration in the vascular tissue. The cascade inflammation is characterized by an instant initial reaction, where the antigen has been detected and damaged that triggers neutrophils to be at the site of reaction; subsequently, the monocytes/macrophages invade this antigen [4]. Inflammation is frequently associated with the initiation and development of cancer, as through inflammation, reactive oxygen and nitrogen species are formed to fight pathogens then promote tissue restoration and regeneration that cause DNA damage, resulting in mutations that promote cancer [5]. 

Inflammation is stimulated by immune cells that produce proinflammatory cytokines that modulate inflammatory reactions. Among these cytokines are interferon-gamma (IFNγ), tumor necrosis factor alpha (TNF-α), as well as interleukin-1β (IL-1β) and IL-6, IL-12, and IL-18 [6]. TNF-α is an inflammatory cytokine that is generated by macrophages/monocytes through acute inflammation and is accountable for several signaling outcomes in cells that result in apoptosis or necrosis [7]. TNF-α also triggers different inflammatory molecules, involving other cytokines and chemokines [8]. IL-1β is a potent pro-inflammatory cytokine needed for the response to any disease and injury. It is produced and released by a various cell types, especially innate immune system cells such as monocytes and macrophages [9]. IL-6 is a multifunctional cytokine that is produced in response to tissue injury and infections [10]. IL-6 is a multifunctional cytokine with broad effects on the immune system; one of its purposes is to sustain immune function. [11]. 

To date, non-steroidal anti-inflammatory drugs (NSAIDs) are the best choice for treating numerous inflammatory diseases [12]. NSAIDs are pain relievers that have anti-inflammatory, antipyretic, fever-reducing, and analgesic properties [1,3,12]. Most NSAIDs have general side effects, mostly gastrointestinal toxicity, because of their free -COOH group [4]. They have also been related to different side effects, including renal injuries, cardiovascular diseases, hypertension, sudden cardiac death, hepatotoxicity, as well as other minor syndromes [13]. NSAIDs act by inhibiting the activity of Cox-enzymes and decreasing prostaglandins in the body, leading to diminished inflammation [14,15]. The adverse effects of NSAIDs are related to the inhibition of COX-1 enzyme, while the desirable antipyretic, anti-inflammatory, and analgesic effects of these medicines are the consequence of COX-2 enzyme inactivation [12,15]. The decrease in prostaglandins, which protect the stomach and maintain platelets and blood coagulation, provide us with the need to find novel anti-inflammatory agents that are safe, low in toxicity, and effective [3]. 

**Figure 1:**
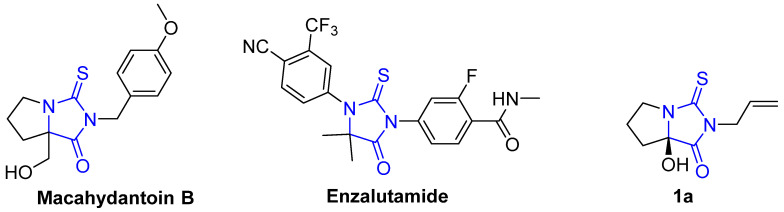
Selected natural and FDA-approved 2-thioxoimidazolidin-4-one analogues.

Over the last few decades, several natural and synthetic heterocyclic compounds have been explored as potential pharmacological pro-drugs [12]. Among these, 2-thiohydantoins (2-thioxo-imidazolidine-4-one), the sulfur analogues of hydantoin, have been recognized as interesting chemical scaffolds because of their occurrence in natural and therapeutically active compounds (Figure 1). Structural alterations in the 2-thiohydantoin ring produce compounds with a wide spectrum of pharmacological and biological effects, including antibacterial, antifungal, human immunodeficiency virus (HIV), antithyroidal, antimutagenic, anticarcinogenic, antiviral, tuberculosis, anti-ulcer and fatty acid amide hydrolase inhibitor [16,17,18,19,20,21,22,23,24]. The 2-thiohydantoin ring has been also incorporated into the structures of several natural products such as Enzalutamide, which has been FDA-approved as a drug for castration-resistant prostate cancer (Figure 1) [25,26,27]. Based on the high therapeutical significance of the 2-thiohydantoin-based compounds, the synthesis of a novel class of substituted-2-thiohydantoins has attracted great attention in recent decades [28,29,30,31,32,33,34,35,36].

**Figure 2:**
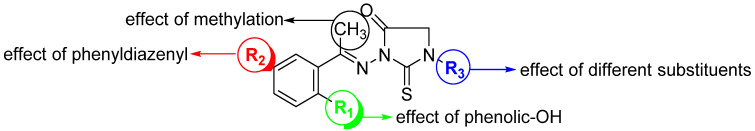
Design of a set of 1,3-disubstituted-2-thioxo-imidazoidin-4-one analogues.

**Figure 3:**
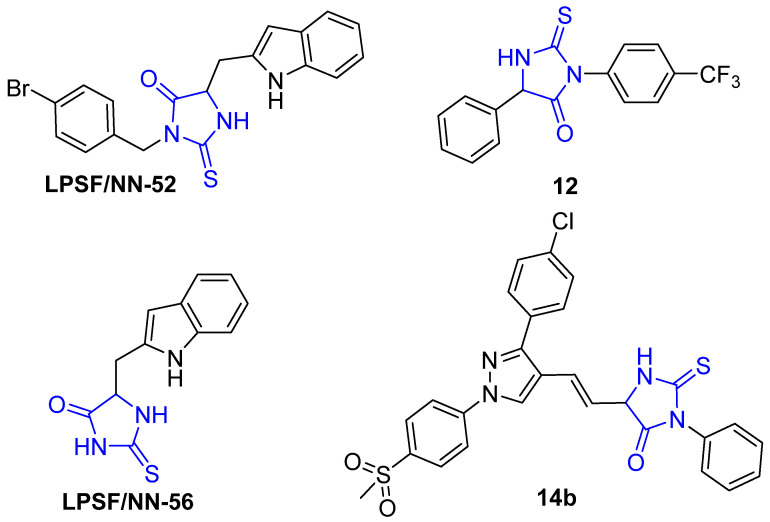
Selected 2-thioxoimidazolidin-4-one analogues with potent anti-inflammatory activity.

Recently, the anti-inflammatory activity of 2-thioxo-imidazolidine-4-ones has been explored and demonstrated by several researchers. Gauthier *et al.* reported the anti-inflammatory activity of a series of 3,5-diaryl-2-thiohydantoins and showed that this class of compounds possesses substantial inhibitory activity toward human recombinant COX-2 (Figure 2, compound **12**). However, these compounds did not exhibit considerable inhibitory activity in LPS-induced blood cells, which were attributed to the lower stability of compounds in aqueous medium [37]. Moreover, da Silva Guerra *et al*. reported the anti-inflammatory activity of 5-substitued-2-thioxoimidazolidin-4-one analogues (LPSF/NN-56) and (LPSF/NN-52), which were previously synthesized by Brandao *et al*. (Figure 3). The authors showed that these compounds reduce the expression of TNF-α and IL-1β and cause a reduction in the migration of leukocyte [4,38]. Recently, Abdellatif *et al*. reported the synthesis of hybrid (3,5-disubstituted)-2-thiohydantoin-pyrazole compounds and showed that this class of compounds exhibits a potent inhibitory activity toward COX-2 enzymes with considerable anticancer activity toward MCF-7, A-549, and HCT-116 cell lines. Furthermore, this class of compounds displays lower ulcerogenic effects than ibuprofen (Figure 3, compound **14b**) [39]. 

The anti-inflammatory activity of 2-thioxoimidazolidin-4-one scaffold was mainly explored for analogues substituted either at position N3- and/or position C5 [4,37,39,40]. As far as we are aware, the anti-inflammatory activity of N1,N3-disubstituted 2-thioxoimidazolidin-4-one has not been explored yet. Molecular hybridization has been considered as one of the most significant strategies in drug design, which includes the combination of different bioactive moieties to afford a novel hybrid structure that exhibits improved efficacy and affinity toward the targeted protein [41,42,43,44]. Schiff bases, including azomethines, have been realized as a potential pharmacophoric moiety [45,46,47,48]. Considering these facts and our interests to synthesize novel bioactive molecules [49,50,51,52,53,54,55,56,57], this study was envisioned to design and synthesize a new set of hybrid 1,3-disubstituted-2-thioxo-imidazolidin-4-one derivatives aiming at investigating different structural features around the 2-thioxo-imidazolidin-4-one scaffold that are responsible for the anti-inflammatory activity. As an attempt to optimize the anti-inflammatory potency of 2-thioxoimidazolidin-4-ones, azomethine moiety was hybrid with 2-thiohydantoin and several substituents and groups have been introduced to the position N1 and N3 of the 2-thioxoimidazolidin-4-one moiety (Figure 2). The anti-inflammatory activity of the designed compounds was assessed by several analyses including cytotoxicity, nitric oxide production, and the expression of different anti-inflammatory cytokines. Finally, a detailed molecular modelling study was performed to explore the binding affinity of this class of compounds toward COX-1 and 2 binding sites. 

## 2. Results and Discussion 

### 2.1. Chemistry

**Scheme 1:**
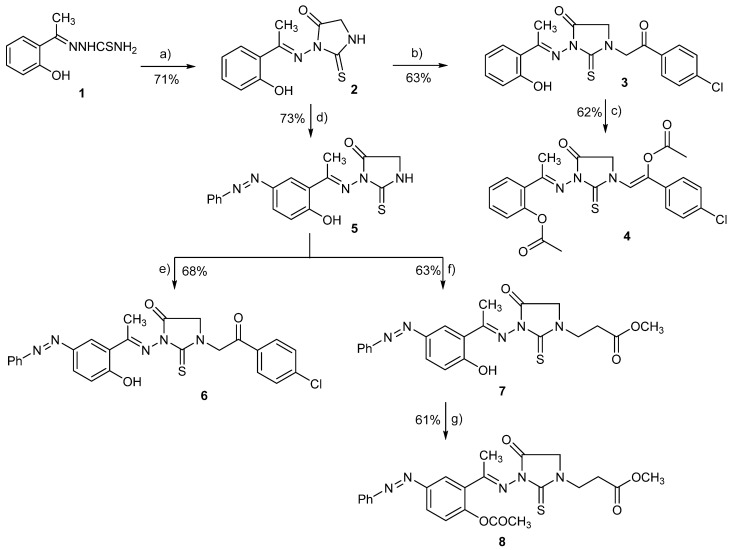
Synthesis of 2-thioxoimidazolidin-4-one derivatives (**2**–**8**). Reagents and reaction conditions: (**a**) Ethylchoroacetate, AcONa, ethanol, reflux, 16 h; (**b**) 4-chlorophenyacyl bromide, Et_3_N, DMF, reflux, 12 h; (**c**) Acetic anhydride, reflux, 16 h; (**d**) PhN≡NCl, NaOH aqu., 0–5 °C, 6 h; (**e**) 4-chlorophenacyl bromide, DMF, Et_3_N, reflux, 12 h; (**f**) Methyl-acrylate, Et_3_N, DMF, reflux, 14 h; (**g**) acetic anhydride, reflux, 16 h.

In this study, a set of novel 1,3-disubstituted-2-thioxoimidazolidin-4-one analogues (**3**–**8**) has been prepared following the synthetic route illustrated in Scheme 1. The synthesis of the main 2-thioxoimidazolidin-4-one scaffold relied on the cyclo-condensation of benzylidenehydrazine-1-carbothioamide to the ethyl chloroacetate as a key reaction step. The synthesis of 1,3-disubstituted-2-thioxoimidazolidin-4-one analogues started from the commercially available 2′-hydroxyacetophenone. Condensation of 2′-hydroxyacetophenone with thiosemicarbazide in the presence of fused sodium acetate afforded 2-[1-(2-hydroxy phenyl) ethylidene] hydrazine-1-carbothioamide (**1**) in quantitative yield [54,58]. Subsequently, compound **1** was subjected to cyclo-condensation with ethyl chloroacetate under reflux in the presence of sodium acetate to successfully provide 3-((1-(2-hydroxyphenyl)ethylidene)amino)-2-thioxoimidazolidin-4-one (**2**) in satisfactory yield (71%) [59,60]. Compound **2** has been then utilized as a main intermediate to successfully achieve the substitution at position 1 and 3 with different substituents in the 2-thioxoimidazolidin-4-one scaffold as illustrated in Scheme 1. Thus, compound **2** was alkylated at position 1 through reaction with 4-chlorophenyacyl bromide under catalytic basic conditions and refluxing to provide 1-[2-(3-chlorophenyl)-2-oxoethyl)-3-[1-(2-hydroxyphenylethylidene)amino]2-thioxoimidazolidin-4-one (**3**) in 63% yield. In order to explore the role of the phenolic-OH in compound activity, compound **3** was further acylated by reaction with acetic anhydride under reflux to furnish 1-[2-(4-chlorophenyl)-2-(acetoxy)ethene-1-yl]-3-[1-(2-acetoxyphenylethylidene)amino]-2-thioxoimidazolidin-4-one (**4**) in a considerable yield (62%). 

To explore further structural features at position 3 of 2-thioxoimidazolidin-4-one scaffold, we have performed an aromatic substitution at position 5 of the (2-hydroxyphenyl) ethylidene] hydrazine moiety with phenyldiazenyl group. Thus, 2-thioxoimidazolidin-4-one (**2**) was reacted with phenyldiazonium chloride at 0–5 °C under basic conditions (10% sodium hydroxide) to afford 3-[1-(2-hydroxy-5-(phenyldiazenyl) phenyl) ethylidene) amino]-2-thioxoimidazolidin-4-one (**5**) in a good yield (73%). Subsequently, compound **5** was utilized to further explore the structural features at position 1 by alkylating the amino group of 2-thioxoimidazolidin-4-one scaffold with different alkylating agents. Toward this, compound **5** was reacted under reflux with 4-chlorophenacyl bromide in the presence of triethylamine to afford 1-[2-(4-chlorophenyl)-2-oxoethyl]-3-[1-(2-hydroxy-5-(phenyldiazenyl) phenyl) ethylidene) amino] 2-thioximidazolidin-4-one (**6**) in a satisfactory yield (68%). Furthermore, compound **5** was reacted with methyl acrylate under basic conditions via Michael addition reaction to furnish 3-{3-[1-(2-hydroxy-5-(phenyl diazenyl) phenyl) ethylidene) amino]-4-oxo-2-thioxoimidazolidine-1-yl}propanoate (**7**) in 63% yield. Finally, the role of the phenolic-OH group was explored by converting into the corresponding acetate. Thus, compound **7** was refluxed with acetic anhydride to provide methyl 3-{3-[1-(2-acetoxy-5-(phenyl diazenyl) phenyl )ethylidene) amino]-4-oxo-2-thioxoimidazolidin-1-yl} propanoate (**8**) in a good yield (61%).

The structure of compounds (**2**–**8**) was determined, characterized and affirmed by several analytical techniques, including melting point, elemental analysis, FT-IR, and nuclear magnetic resonance analysis (^1^H- and ^13^C-NMR). The ^1^H-NMR spectra of compound **2** revealed four singlet signals at δ 12.60, 12.16, 4.02, and 2.54, ppm, which were assigned to the protons of phenolic hydroxyl (OH), NH-group, methylene group of the imidazolidine ring, and methyl group in the hydrazinyl side chain at position-3, respectively. The ^1^H-NMR spectra also showed the aromatic protons resonate as characteristic multiple signals. The ^13^C-NMR spectra exhibited signals of the imidazolidine ring at δ 174.04, 166.44, and 33.99 ppm, which has been assigned to (C=S), (C=O), and methylene group, respectively. These results affirm the successful cyclo-condensation and the formation of 3-substituted-2-thioximidazolidin-4-one scaffold. The ^1^H-NMR spectra of compound **3** revealed that this compound presents in a tautomeric equilibrium (keto-enol tautomer) (Figure S1, Supporting information). The ^1^H-NMR spectra showed a singlet signal at δ 12.16 ppm which assigned to the OH-proton of the enol form, together with a singlet signal at δ 2.16 ppm, which referred to the olefinic proton (−CH=C−OH). The protons of the methylene group in the imidazolidine ring (N−CH_2_CO) were observed at δ 5.47 ppm. The ^13^C-NMR spectra of compound **3** displayed two carbon signals at δ 192.67 and 66.84 ppm, assigned to the −CH_2_CO− group. On the other hand, the two carbon signals at δ 170.35 and 20.82 ppm were referred to the carbon signals of the (−CH=C−OH) group in the of enol-form (Figure S1a,b, Supporting information). The ^1^H-NMR spectra of diacetoxy derivative **4** affirmed the absence of proton signals at δ 12.60–12.16 and 5.47 ppm, which referred to the two OH-group and the methylene group. The appearance of two new characteristic singlet signals at δ 2.32 and 2.23 ppm was attributed to the two acetyl (COCH_3_) groups. The protons of methylene in the imidazole ring appeared at δ 4.25–4.40 ppm. The observed quartet signal for these protons could be attributed to the interaction and split by the methyl protons of the acetyl group (COCH_3_). The olefinic and aromatic protons (CH=CH) were displayed at δ 7.12–8.03 ppm as multiple signals. The ^13^C-NMR spectra showed four carbon signals at δ 164.60, 162.93, and 22.12, 21.24 ppm assigned to the two acetyl (2× COCH_3_) groups. The olefinic carbon signal (CH=C−) was also observed at δ 80.26 ppm. The C−O and aromatic ring carbon signals were displayed at δ 151.28–124.62 ppm. Furthermore, the ^13^C-NMR spectra displayed three carbon signals at δ 186.65, 169.17 and 43.55 ppm, which was attributed to (C=S), (C=O), and (CH_2_) of the imidazole ring, respectively (Figure S2a,b, Supporting information). 

The ^1^H-NMR spectra of compound **5** showed four characteristic singlet signals at δ 13.31, 12.25, 4.05, and 2.65 ppm referred to as the hydroxyl (OH), amino (NH), methylene protons (CH_2_), and methyl protons (CH_3_), respectively. The aromatic ring protons were displayed in the characteristic regions at δ 7.13–8.27 ppm as doublet and multiple signals. The ^13^C-NMR spectra of compound **5** exhibited three carbon signals at δ 174.09, 166.07, and 34.10 ppm, which were attributed to the (C=S), (C=O), and (CH_2_) groups of the imidazole ring. The carbon signal of the methyl group (CH_3_) was displayed at δ 14.82 ppm. The characteristic carbon signals of the aromatic rings and C=N appeared at δ 164.42–118.71 ppm (equal 13 carbon atoms) (Figure S3a,b, Supporting information). The ^1^H NMR spectra of compounds **6** displayed proton signals at δ 5.38, 7.69–7.71, and 8.13–8.15 ppm, which were attributed to the aromatic ring protons and the methyl (CH2-CO) of the 4-chlorobenzoylmethyl moiety. The ^13^C-NMR spectra indicated the presence of compound **6** in two stereoisomers (E/Z isomers) which was represented by the two carbon signals at δ 197.45, 191.36 ppm for the carbonyl group (C=O), two carbon signals at δ 56.51, 49.48 ppm for the methylene group, two carbon signals at δ 33.30, 27.22 ppm for the (CH_2_) group of the imidazole ring, and two carbon signals at δ 19.04 and 14.76 ppm for the methyl (CH_3_) group (Figure S4a,b, Supporting information). The ^1^H-NMR spectra of compound **7** displayed three new proton signals at δ 3.99–4.02 ppm as a triplet, δ 3.62 as a singlet, and δ 2.73–2.76 ppm as a triplet signal, which were attributed to the (NCH_2_), (OCH_3_), and (CH_2_CO) groups, respectively, of the N−CH_2_CH_2_COOCH_3_ moiety. The remaining characteristic proton signals in compounds **7** were displayed in the expected chemical shift regions (Figure S5a,b, Supporting information). The ^1^H-NMR Compound **8** indicated the absence of a singlet signal at δ 13.18 ppm which was attributed to the phenolic-OH group, confirming the acylation of compound **8**. Furthermore, a new singlet signal at δ 1.78 ppm for the acetyl protons (COCH_3_) also appeared. The protons of the two methylene groups (CH_2_) were shown as triplet signals at δ 4.03 and 2.76 ppm, while the protons of methylene (CH_2_) of the imidazole ring were displayed as characteristic singlet signal at δ 4.09 ppm. The protons of the methoxy (OCH_3_) and methyl (CH_3_) groups were observed as singlet signals at δ 3.62 and 2.65 ppm, respectively. Furthermore, the ^1^H-NMR spectra displayed the aromatic proton signals (CH=CH) at δ 7.08–8.24 ppm with characteristic multiplicity (Figure S6a,b, Supporting information).

### 2.2. In Vitro Assessment of Cytotoxic Activity against LPS-Activated RAW264.7 Cells

**Figure 4:**
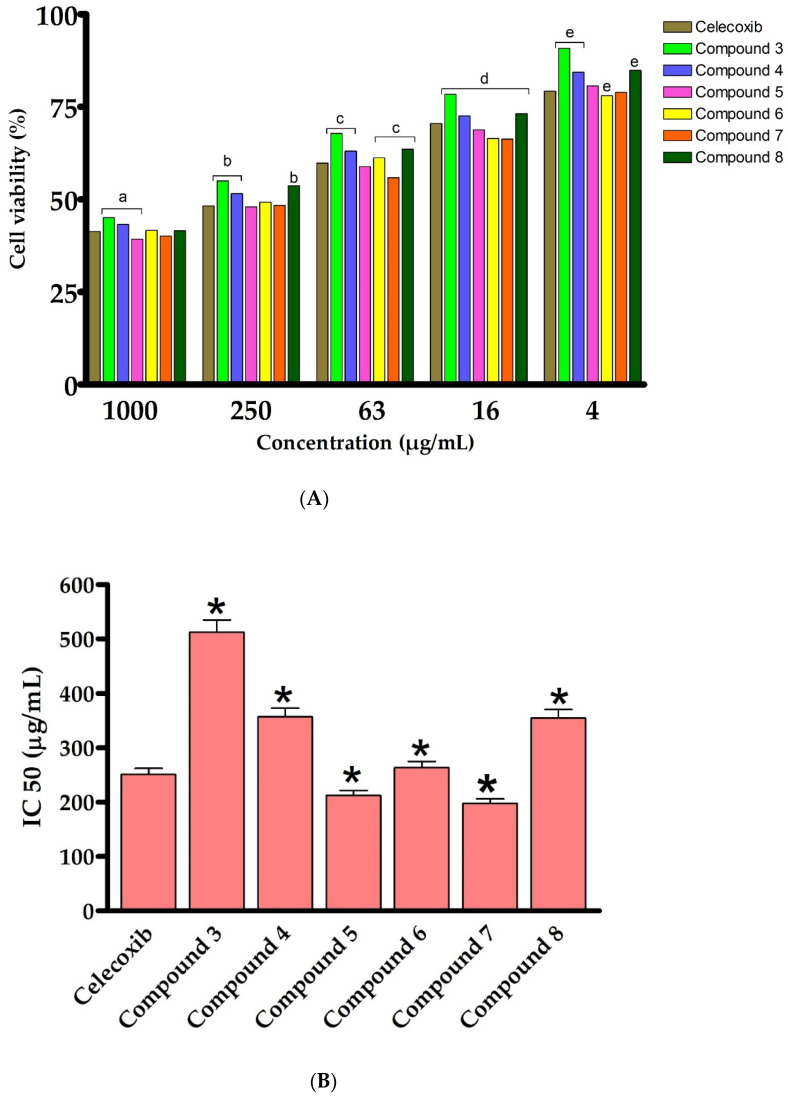
In vitro cytotoxic effect of 2-thioxoimidazolidin-4-one derivatives (**3**–**8**) against murine cell line (RAW264.7). (**A**): cell viability of celecoxib and compounds **3–8** at different concentrations. (**B**): IC_50_ (μg/mL) against RAW264.7. Data are shown as mean ± SD. ^a^ *p* < 0.05 versus celecoxib at concentration of 1000 μg/mL, ^b^ *p* < 0.05 versus celecoxib at concentration of 250 μg/mL, ^c^ *p* < 0.05 versus celecoxib at concentration of 63 μg/mL, ^d^ *p* < 0.05 versus celecoxib at concentration of 16 μg/mL, ^e^ *p* < 0.05 versus celecoxib at concentration of 4 μg/mL and * *p* < 0.05 versus celecoxib.

We have first assessed the cytotoxic activity of the synthesized 2-thioxoimidazolidin-4-one derivatives (**3**–**8**) on an LPS- activated murine RAW264.7 cell line, utilizing the MTT assay. Toward this, cells were initially treated with LPS (5 μg/mL), which activated and stimulated cells (monocyte/macrophage) to produce a variety of pro-inflammatory factors and induce inflammation [61,62,63]. The cells were then exposed for 24 h to the synthesized analogues (**3**–**8**) at different concentrations (1000, 250, 63, 16 and 4 μg/mL). In our assessments, celecoxib, a nonsteroidal anti-inflammatory drug (NSAID) and inhibitor of cyclooxygenase-2 (COX-2), was employed as a positive reference anti-inflammatory drug. As outlined in Figure 4, the results showed that, except for compound **3**, compounds **4**–**8** exhibited a high cytotoxicity against LPS-activated RAW264.7 cells at different concentrations (1000, 250, 63, 16 and 4 μg/mL). The introduction of the 4-chlorophenyacyl group at position N1 provided a 1,3-disubstituted-2-thioxoimidazolidin-4-one analogue (**3**), which has considerable cytotoxic activity (IC_50_ 500 μg/mL). Acylation of compound **3** noticeably improved the cytotoxic activity of the compound, as revealed for compound **4** (IC_50_ 355 μg/mL), suggesting the role of the acetyl groups in the cytotoxic activity of compound **4**. To explore the structural features at position N3 of 2-thioxoimidazolidin-4-one scaffold, an aromatic substitution with phenyldiazenyl group was performed at position N5 of the (2-hydroxyphenyl) ethylidene] hydrazine moiety. Interestingly, the introduction of the phenyldiazenyl group at position 5 (compound **5**) significantly improved the cytotoxicity of the compound (IC_50_ 212.3 μg/mL). Based on these results, compound **5** was utilized as a scaffold to introduce different groups at position N1. To this end, the introduction of 4-chlorophenyacyl group did not provide a meaningful effect on the cytotoxic activity of the compound (compound **6**, IC_50_ 263 μg/mL). On the other hand, the alkylation of N1 with methyl-proponate group (Compound **7**) resulted in a considerable improvement in the cytotoxicity of the compound (IC_50_ 197.7 μg/mL). Acylation of compound **7** significantly reduced the cytotoxic activity of compound (IC_50_ 354 μg/mL), implying the role of the phenolic hydroxyl group in the cytotoxic activity of compound **7**. These results are in agreement with other studies which demonstrated that 2-thioxoimidazolidin-4-one analogues possess a potent anti-inflammatory activity [4,37,39,40]. Celecoxib is a NSAID which is used to treat rheumatoid arthritis, pain, and inflammation. However, it possesses a set of side effects, mainly gastrointestinal problems. The discovery of new effective anti-inflammatory agents, thus, considered as an urgent concern [4,5]. Several reports demonstrated 2-thioxoimidazolidine-4-one analogues as a potent class of compounds which possess a broad-spectra of pharmacological and biological activities [64,65,66]. Among synthesized and evaluated compounds, compound **5** and **7** exhibited the most potent cytotoxic activity against LPS-activated RAW264.7 cells (IC_50_ of 212.3 μg/mL and 197.68 μg/mL, respectively), comparable to that of celecoxib drug (IC_50_ value 251.2 μg/mL) (Figure 4). These findings indicate that 1,3-disubstituted-2-thioxoimidazolidin-4-one could be considered as a promising class of potent anti-inflammatory agents.

### 2.3. In Vitro Evaluation of the Anti-Inflammatory Activity against LPS-Activated RAW264.7 Cells by Assessing NO Production

**Figure 5:**
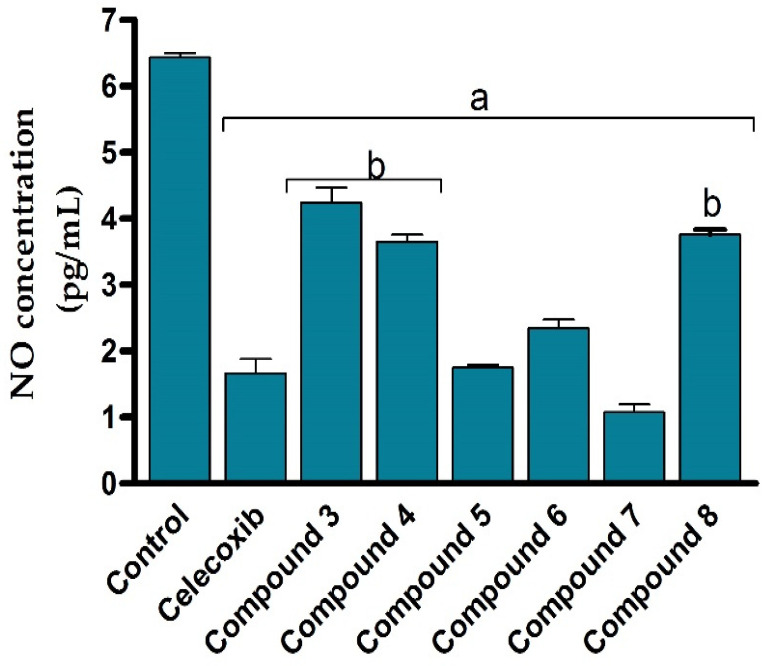
Anti-inflammatory effects of 2-thioxoimidazolidin-4-one derivatives (**3**–**8**) against LPS-induced murine cell line (RAW264.7) by evaluating NO production. Data are shown as mean ± SD. ^a^ *p* < 0.05 versus control cells and ^b^ *p* < 0.05 versus celecoxib.

Encouraged by the potent cytotoxic activity of synthesized compounds, we next assessed the anti-inflammatory activity of the compounds (**3**–**8**) against LPS-activated RAW264.7 cells by evaluating the production of nitric oxide. Toward this aim, RAW264.7 cells were subjected to LPS (5 μg/mL) to induce the production of the inflammatory mediators such as nitric oxide. The cells were then treated with different concentrations of the synthesized 2-thioxoimidazolidin-4-one derivatives (**3**–**8**) and the production of nitrile was colourimetrically assessed as an indicator for NO production. In our assessments, celecoxib was applied as a reference anti-inflammatory drug. As shown in Figure 5, the NO production assay results revealed that all 2-thioxoimidazolidin-4-one derivatives (**3**–**8**) exhibit considerable anti-inflammatory activity against LPS-activated RAW264.7 cells via significantly (*p* < 0.05) suppressing nitric oxide release, compared to the control LPS-activated RAW264.7 cells. Interestingly, the anti-inflammatory activity of the compounds (**3**–**8**) was in harmony with the cytotoxic activity. The structural features responsible for the cytotoxic activity of the compounds were in accordance with the inhibitory activity of the compounds to the production of the inflammatory reactive nitric oxide. For instance, the introduction of the 4-chlorophenyacyl group at N1 position provided a 1,3-disubstituted compound with moderate anti-inflammatory activity (compound **3**), while the acylation of this compound showed considerable improvement in the activity (compound **4**). Furthermore, the aromatic substitution with phenyldiazenyl group at position N5 of the (2-hydroxyphenyl) ethylidene] hydrazine moiety afforded a compound with anti-inflammatory activity similar to that of celecoxib. While the introduction of the 4-chlorophenyacyl group at N1 position showed no significant effect on anti-inflammatory activity (compound **6**), the introduction of the methyl-proponate group demonstrated a substantial effect on anti-inflammatory activity (compound **7**). Finally, the acylation of compound **7** significantly diminished the activity of the compound, affirming the role of the phenolic group on the anti-inflammatory activity of the compound. Treatment of cells with LPS stimulated ERK, JNK, and p38 phosphorylation to induce the expression of inflammatory mediators and cytokines by promoting the production of nitric oxide, interleukin (IL)-6, and tumor necrosis factor alpha (TNF-α) in RAW264.7 cells [67,68]. Nitric oxide is an unstable and toxic molecule that spontaneously transferred into a stable nitrite/nitrate format. The diminished concentration of nitric oxide in LPS-stimulated RAW264.7 cells to a normal value revealed that all synthesized 2-thioxoimidazolidin-4-one derivatives (**3**–**8**) possess substantial anti-inflammatory activity to reduce the production of inflammatory reactive nitrogen species in LPS-activated RAW264.7 cells [69]. Among different compounds, compound **7** demonstrated the most potent inhibitory activity toward the production of nitric oxide on LPS-activated RAW264.7 cells, suggesting that compound **7** could be considered as a promising anti-inflammatory agent.

### 2.4. Assessment of IL-1β Expression (Western Blot Analysis) 

**Figure 6:**
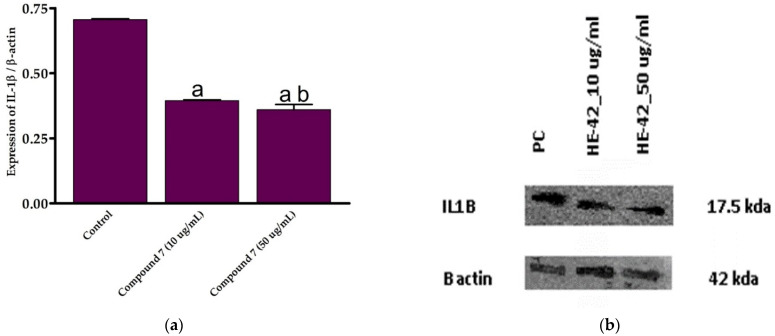
*In vitro* anti-inflammatory effect of compound **7** against LPS-activated RAW264.7 cell line. (**a**) Expression of IL-1β at different concentration of compound **7**; (**b**) Western plot analysis of the expression of IL-1β. Data are shown as mean ± SD. ^a^ *p* < 0.05 versus control cells and ^b^ *p* < 0.05 versus compound **7** (10 μg/mL).

Based on the assessment of the cytotoxicity and anti-inflammatory activities, compound **7** was selected for further examinations. To explore the mechanistic insights into the anti-inflammatory activity of this class of compounds, we estimated the expression of IL-1 β in LPS-activated RAW264.7 cells at different concentrations of compound **7** (10 and 50 μg/mL) by Western blot analysis, following normalization to β-actin protein. IL-1β is a critical pro-inflammatory cytokine that enhances adhesion molecule production and leukocyte movement. The cytokine IL-1β is a member of the IL-1 peptide group that plays a vital role in tumor formation and inflammatory activation. Inflammatory stimuli such as LPS and pro-inflammation IL-1β cytokine cause a dramatic upregulation in the reactive oxygen species (ROS) production, which alters several cellular activities, including transcription factor activation, DNA synthesis, proliferation, and gene expression [4,68,69]. In the current study, we were interested in exploring whether the anti-inflammatory activity of compound **7** could be due to its ability to reduce the expression of pro-inflammatory IL-1β cytokine. To this end, the effect of compound **7** on the expression of IL-1β was assessed at different concentrations. Our results demonstrated that compound **7** (10 and 50 μg/mL) significantly reduced (*p* > 0.05) LPS-induced IL-1β expression by 0.395 and 0.341 folds, respectively, compared to control cells (Figure 6). Moreover, the 50 μg/mL concentration of compound **7** significantly suppressed (*p* > 0.05) IL-1β expression (Figure 6). These findings indicate that compound **7** at a concentration of 50 μg/mL exhibits a potent anti-inflammatory effect via diminishing pro-inflammatory IL-1β cytokine expression. 

### 2.5. Assessment of IL-6 and TNF-α Expression

**Figure 7:**
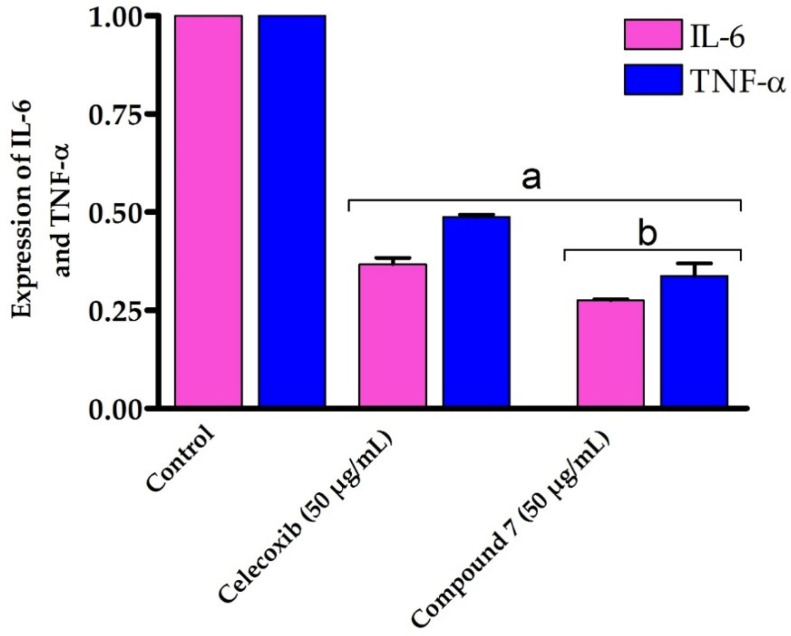
*In vitro* anti-inflammatory effect of compound **7** against LPS-activated RAW264.7 cell line on IL-6 and TNF-α expression. Data are shown as mean ± SD. ^a^ *p* < 0.05 versus control cells and ^b^ *p* < 0.05 versus celecoxib.

Our results encouraged us to further study the mode of action accounting for the potent anti-inflammatory activity of compound **7**. Toward this, we have evaluated the effect of compound **7** on the expression of the inflammatory cytokines IL-6 and TNF-α by RT-PCR. TNF-α is an effective cytokine that stimulates the inflammation mechanism and triggers various inflammatory diseases [69]. IL-6 is excreted by several cells in response to the inflammation process and plays a critical pathological role in autoimmunity and chronic inflammation [70,71,72]. Several studies have shown that the expression of IL-6 and TNF-α is dramatically upregulated in cells treated with LPS [73,74]. In our investigations, RAW264.7 cells were treated with LPS to induce the production of inflammatory cytokines, followed by the addition of compound **7** (50 μg/mL). The 50 μg/mL dose was selected as it demonstrated the most potent anti-inflammatory effect by downregulating the expression of the pro-inflammatory IL-1β cytokine. After 48 h, cells were gathered and the expression of IL-6 and TNF-α was assessed by RT-PCR and compared to either control cells or celecoxib-treated cells (50 μg/mL) as reference anti-inflammatory drug. As shown in Figure 7, compound **7** significantly downregulated (*p* > 0.05) the expression of both IL-6 and TNF-α genes on LPS-activated RAW264.7 cells by 0.276 and 0.314 folds, respectively, compared with control cells. Interestingly, compound **7** exhibited the ability to reduce the expression of IL-6 and TNF-α genes compared to celecoxib-treated LPS-activated RAW264.7 cells. Our findings are in accordance with several studies which showed that hydantoins, analogues of thiohydantoins, are potent anti-inflammatory agents by inhibiting TNF-α precursor enzyme [4,5,75]. Our results indicate that compound **7** exhibits an efficient anti-inflammatory activity against LPS-activated RAW264.7 cells by suppressing IL-6 and TNF-α cytokines.

**Figure 8:**
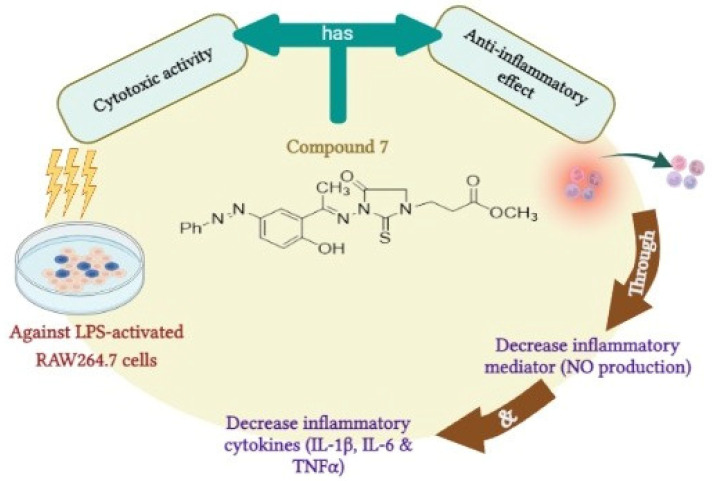
The predicted mode of action for the anti-inflammatory activity of compound **7**.

Taken together, our findings suggest that compound **7** could be considered as a lead 1,3-disubstituted-2-thioxoimidazolidin-4-one analogue that may be utilized for the development of potent anti-inflammatory agents. Our study showed that compound **7** possesses potent anti-inflammatory and immunomodulatory effects via different mechanisms including the high cytotoxicity against LPS-activated RAW264.7 cells, the ability to significantly inhibit the production of nitric oxide, and the down regulation of inflammatory cytokines, including IL-1β, IL-6, and TNF-α (Figure 8). The potent and wide-range anti-inflammatory mode of action could be applicable to various inflammatory reactions. Nevertheless, further studies should be performed to explore the bio-applicability and the anti-inflammatory activity of compound **7** in in vivo research.

### 2.6. Assessment of Binding Affinity toward COX-1 and COX-2 

**Figure 9:**
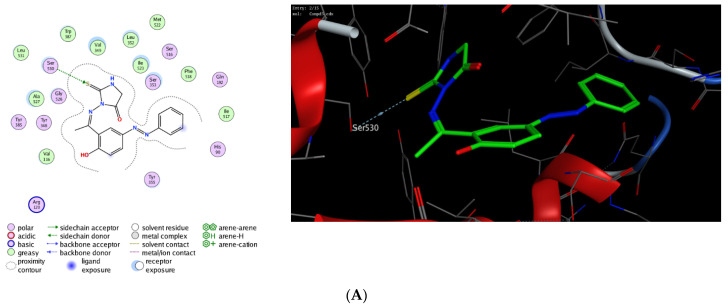

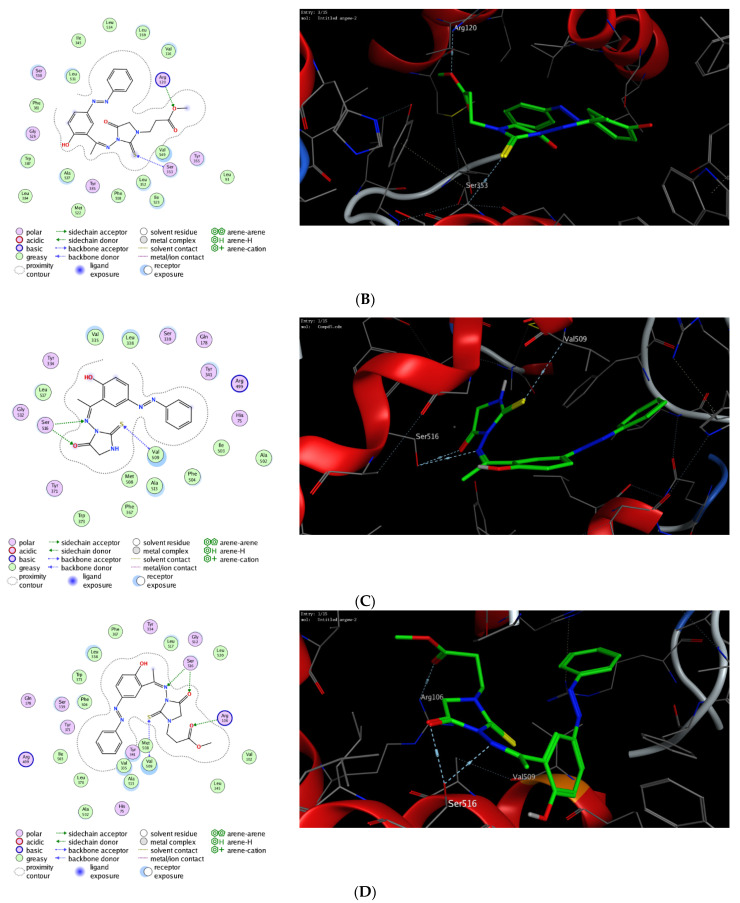
Interactions (2D and 3D) of compound **5** (**A**,**C**), and compound **7** (**B**,**D**) into the binding site of COX-1 (PDB code: *3kk6*) and COX-2 (PDB code: *3ln1*) proteins.

The molecular modelling technique affirms the power to assess the binding affinity of bioactive molecules toward the binding pocket of a targeted protein and offers unique information to justify the mode of action of pharmacological compounds [76,77,78,79,80]. Toward this, we were interested in exploring whether the observed potent anti-inflammatory activity of this class of compounds could be due to their ability to target Cyclooxygenases enzymes. Cyclooxygenase exists in two isoforms (COX-1 and COX-2) that are mainly responsible for the production of prostaglandin. While COX-1 is expressed in several tissues, the expression of COX-2 is mainly overexpressed in various cell types by several inducers including hormones, growth factors, cytokines, and lipopolysaccharide [81,82,83,84,85]. Several studies have indicated that the required anti-inflammatory, antipyretic and analgesic activities of NSAID are mainly due to the inhibition of COX-2 activity, whereas the adverse effects of these drugs is assigned to the inactivation of COX-1 activity [86,87,88]. Furthermore, it has been shown that COX-2 is overexpressed in several neurodegenerative diseases, angiogenesis, and cancer [89,90,91,92]. Thus, the discovery of a new class of anti-inflammatory agents, which is selectively targeting COX-2 activity, is urgently needed. In this regard, extensive molecular docking studies were performed aiming at evaluating the binding affinity of this class of compounds toward the active site of COX-1 and 2 enzymes, compared to the co-crystallized celecoxib drug. To examine the binding mode of mono *N*3-substituted- and *N*1, *N*3-disubstituted-2-thiohydantoins, compounds **5** and **7** were selected as representative compounds for this class of compounds to explore their binding affinity toward the active pocket of COX1 and COX-2 enzymes (PDB codes: *3kk6* and *3ln1*, respectively). The applied protocol was first examined for the validity by docking the celecoxib drug (co-crystallized drug) into the active site of COX-1 and 2 enzymes to affirm that celecoxib, under the applied system, can form the original interactions reported in the crystal structure. Next, the protocol was utilized to perform the molecular modelling study of compound **5** and **7** and the obtained data were analyzed to estimate the binding score and binding modes. As shown in Table 1, compound **5** and **7** demonstrated a considerable binding affinity toward the cyclooxygenase enzymes. Both compounds (**5** and **7**) exhibited thermodynamically favorable interactions toward COX-1 and 2 binding sites, as indicated by the negative values of affinity scores. Toward COX-1, compound **5** displayed moderate binding affinity toward the binding cavity via forming a hydrophilic interaction through its thio-carbonyl group with Ser530 amino acid residue (−8.36 kcal/mol). On the other hand, compound **7** exhibited a higher binding affinity toward COX-1 cavity (−9.54 kcal/mol). As indicated in Figure 9, compound **7** demonstrated the ability to form a similar hydrophilic interaction as compound **5** with Ser353 amino acid residue, but also an additional interaction with Arg120 residue via the methyl-poropyl ester group in the N1 position. Furthermore, compound **7** showed the possibility to stabilize the binding pose via hydrophilic interaction with a set of greasy residues in the pocket. These results indicate that the N1,N3-disubstitution-scaffold has a better binding affinity that the mono-substituted-2-thiohydantoin. Regarding the COX-2 enzyme, compound **5** and **7** displayed considerable binding affinity toward the binding pocket of the COX-2 enzyme (−9.96 and −11.17 kcal/mol, respectively) via interacting hydrophilically and hydrophobically with a set of amino acid residues. As shown in Table 1, compound **5** showed the ability to bind to the COX-2 binding site via its 2-thiohydantoin moiety to two amino acid residues (Ser516 and Val509). Furthermore, the azomethine moiety displayed hydrophilic interaction with the Ser516 residue. The most stable pose for compound **5** showed that the hydrophilic interactions play a critical role in the stability and binding affinity of the compound. Like compound **5**, compound **7** displayed similar hydrophilic interactions with Ser516 and Val509 amino acid residues, but also the ability to interact with Arg106 residue via its methyl-propyl moiety at N1-position. Over again, these results affirm that the di-substituted-2-thiohydantoin scaffold binds with higher binding affinity to the active site of COX-2 enzyme. Together, our molecular modelling studies indicate that the anti-inflammatory activity of this class of compounds could be due to their high binding affinity toward COX-enzymes, and that 1,3-disubstituted-thoihydantoin (compound **7**) has potent affinity toward COX-2 than COX-1 binding pocket.

## 3. Materials and Methods

### 3.1. General Description of Instrumentation and Reagents

The reagents utilized in this study have been acquired from Sigma Aldrich (St. Louis, MO, USA), Alfa Aesar (Haverhill, MA, USA), or TCI company (Gurugram, India) and were of high-grade quality (>95%). The solvents utilized in the current study were distilled before they were used. The reaction was followed up utilizing thin layer chromatographic (TLC) analysis. The TLC analysis was also applied to ensure the purity of the final product to homogeneity. The TLC plates utilized in this study were aluminum sheets pre-coated with silica gel (Merck KGaA, Darmstadt, Germany) and the spots were identified by illumination. All compounds were characterized by several analytical methods including melting point, elemental analysis, Fourier-transform infrared analysis, and nuclear magnetic resonance analysis. The melting point was assessed utilizing an electrothermal 9200 numerical melting point system (Avantor, Radnor Township, PA, USA). A Perkin-Elmer CHN-elemental analyzer (Perkin-Elmer, Waltham, MA, USA) has been utilized to perform elemental analysis and were realized within ±0.4% of the calculated value. An avatar series FT-IR spectrophotometer (Perkin-Elmer, Waltham, MA, USA) was used to record the infrared spectra in the KBr disc. The nuclear magnetic resonance spectra (^1^H- and ^13^C-NMR) were recorded as a solution in DMSO-*d6*, utilizing a DPX-400 spectrometer (Bruker, Billerica, MA, USA) operating at 400 MHz for ^1^H-NMR and 100 MHz for ^13^C-NMR at 27 °C. The chemical shifts were recorded in δ-scale with reference to the internal standard TMS. The coupling constant (*J*) was recorded in Hz. 

### 3.2. Synthetic Procedures and Analytical Data

#### 3.2.1. Synthesis of 3-[1-(2-Hydroxyphenylethylidene) amino]-2-thioxoimidazolidin-4-one (**2**)

A stirred solution of thiosemicarbazone derivative **1** (2.1 g, 0.01 mol) in absolute ethanol (30 mL) was treated with anhydrous sodium acetate (2.45 g, 0.03 mol), followed by ethyl chloroacetate (1.25 g, 0.01 mol). The resulting reaction mixture was allowed to reflux for 12 h, until TLC analysis revealed a complete reaction. The reaction mixture was poured into ice-cold water with stirring. The resultant precipitate was isolated by filtration, washed with water, and dried. The obtained crude product was further purified by recrystallization in ethanol to obtain compound **2** as yellow crystals. R*_f_*: 0.34 (ethylacetate 100%, visualized with UV illumination). Yield: 71%, m.p. 225 °C. FT-IR (KBr)γ_max_: 3421 (br.OH), 3221 (NH), 1693 (C=O), 1631 (C=N), 1065, 1033 (C−O) cm^−1^. ^1^H-NMR (DMSO-*d6*, 400 MHz, ppm): δ 2.52, 2.54 (s, 3H, CH_3_), 4.02 (s, 2H, CH_2_ of imidazole ring), 6.92–6.99 (m, 2H, Ar-H), 7.32–7.36 (t, 1H, Ar-H, one isomer), 7.40–7.43 (t, 1H, Ar-H, second isomer), 7.65–7.67 (d, 1H, Ar–H, one isomer), 7.77–7.79 (d, 1H, Ar-H, second isomer), 12.16 (s, 1H, NH), 12.60 (s, 1H, OH), 12.98 (s, 1H, SH). ^13^C-NMR (DMSO-*d6*, 100 MHz, ppm): δ 174.0 (C=S), 168.4, 166.4 (C=O), 163.6 (C=N), 160.1, 159.5 (C−O), 133.2, 132.3, 130.2, 129.6, 119.8, 119.6, 119.5, 119.4, 117.7, 117.5 (C-aromatic of two isomer), 44.0 (CH_2_ of imidazole ring), 15.4, 14.8 (CH_3_ of two isomer). Anal. Calcd. for C_11_H_11_N_3_O_2_S (MWT = 249): C, 53.01; H, 4.42; N, 16.87. Found: C, 52.88; H, 4.17; N, 16.66.

#### 3.2.2. Synthesis of 1-[2-(4-Chlorophenyl)-2-oxoethyl]-3-[1-(2-hydroxyphenylethylidene) amino]-2-thioxoimidazolidin-4-one (**3**)

To a stirred solution of imidazolidine-4-one derivative **2** (2.5 g, 0.01 mol) in dimethylformamide (25 mL) was added triethylamine (3 g, 0.03 mol), followed by 4-chlorophenyacyl bromide (2.3 g, 0.01 mol). The resulting reaction mixture was heated under reflux and followed by TLC analysis. After being stirred in the same conditions for 12 h, the TLC analysis showed a compete reaction. The resulting mixture was cooled to an ambient temperature and the reaction was quenched with water. The resultant mixture was neutralized with aqueous HCl solution (2%) and the obtained solid was isolated by filtration. The crude solid was then washed with water and dried. Finally, recrystallization of resultant solid with ethanol successfully afforded compound **3** as yellow crystals. R*_f_*: 0.64 (PE: ethylacetate 2:8, visualized with UV illumination). Yield: 63%, M.p. 193 °C. FT-IR (KBr)γ_max_: 3420 (br.OH), 1705–1691 (br.C=O), 1625 (C=N), 1605, 1588 (C=C), 1081, 1036 (C−O) cm^−1^. ^1^H-NMR (DMSO-*d6*, 400 MHz, ppm): δ 2.16 (s, 1H, H-olefinic), 2.55 (s, 3H, CH_3_), 4.03 (s, 2H, CH_2_ of imidazole ring), 5.42 (s, 2H, CH_2_CO), 6.92–6.96 (t, 2H, Ar-H), 7.33–7.37 (t, 1H, Ar-H), 7.66–7.68 (d, 1H, Ar-H), 7.79–7.86 (m, 1H, Ar-H), 7.90–7.92 (d, 1H, Ar-H), 8.00–8.02 (d, 1H, Ar-H), 12.16 (br-s, 1H, OH), 12.60 (s, 1H, OH). ^13^C-NMR (DMSO-*d6*, 100 MHz, ppm): δ 192.7, 186.6 (C=O of ketone), 174.1 (C=S), 170.4, 166.5 (C=O), 163.6 (C=N), 159.5 (C−O), 133.3, 132.5, 132.0, 131.9, 131.8, 130.4, 130.3, 129.3, 128.5, 119.6, 119.4, 117.5 (C-Aromatic of two isomer), 66.8 (NCH_2_CO), 43.5 (=C−OH), 33.9 (CH_2_ of imidazole ring), 28.2 (CH=C, CH olefinic), 14.8 (CH_3_). Anal. Calcd. for C_19_H_16_N_3_ClO_3_S (MWT = 401.5): C, 56.79; H, 3.98; N, 10.46. Found: C, 56.53; H, 3.71; N, 10.22.

#### 3.2.3. Synthesis of 1-[2-(4-Chlorophenyl)-2-(acetoxy) ethen-1-yl]-3-[1-(2-acetoxyphenyl ethylidene) amino]-2-thioxoimidazolidin-4-one (**4**)

Compounds **3** (4 g, 0.01 mol) was dissolved in acetic anhydride (40 mL) and the resulting solution was refluxed for 16 h. After the TLC analysis indicated a complete acylation, the reaction mixture was cooled to an ambient temperature and then poured into ice-H_2_O. The resultant mixture was allowed to stand for 24 h at 4 °C, during which yellow crystals precipitated. The mixture was subsequently filtered, washed with water, and dried. Finally, the obtained crude product was re-crystallized, utilizing ethanol as a solvent to afford compound **4** as yellow crystals. R*_f_*: 0.49 (PE: ethylacetate 3:2, visualized with UV illumination). Yield: 62%, m.p. 208 °C. FT-IR (KBr)_γ max_: 1752–1691 (C=O), 1629 (C=N), 1605, 1581 (C=C), 1083, 1035(C-O) cm^−1^. ^1^H-NMR (DMSO-*d6*, 400 MHz, ppm): δ 1.94 (s, 3H, CH_3_), 2.23 (s, 3H, COCH_3_), 2.32 (s, 3H, COCH_3_), 4.25–4.40 (q, 2H, CH_2_ of imidazole ring), 7.12–7.14 (d,1H, Ar-H), 7.30–7.34 (t, 1H, Ar-H), 7.42–7.46 (t, 1H, Ar-H), 7.74–7.76 (d, 1H, Ar-H), 7.83–7.87 (t, 2H, Ar-H), 8.01–8.03 (d, 1H, Ar-H). ^13^C-NMR (DMSO-*d6*, 100 MHz, ppm): δ 186.0 (C=S), 169.2, 164.6 (C=O), 162.9 C=N), 151.3, 148.9 (C-O), 132.7, 132.0, 131.9, 131.8, 130.7, 130.4, 129.9, 129.6, 129.3, 125.9, 124.6, (C-Aromatic), 80.3 (CH-olefinic), 43.6 (CH_2_ of imidazole ring), 22.1, 21.2 (2× COCH_3_), 14.6 (CH_3_).

#### 3.2.4. Synthesis of 3-[1-(2-Hydroxy-5-(phenyl diazinyl) phenyl ethylidene)-amino]-2-thioxoimidazolidin-4-ones (**5**) 

To a stirred solution of compound **2** (2.5 g, 0.01 mol) in an aqueous sodium hydroxide solution (10 mL, 10%) at 0–5 °C was added a pre-cooled solution of phenyl diazonium salt (1 g, 0.01 mol) dropwise with stirring for 15 min. The resulting reaction mixture was allowed to stir for additional 60 min at the same conditions, before it was allowed to stand for 6 h at 4 °C. The resultant precipitation was filtered, washed with water, dried, and purified by recrystallization using ethanol as a solvent to yield compound **5** as orange crystals. R*_f_*: 0.38 (PE: ethylacetate 1:9, visualized with UV illumination). Yield: 73%, m.p. 270 °C. FT-IR (KBr)_γmax_: 3420 (br.OH), 3218 (NH), 1689 (C=O), 1625 (C=N), 1605, 1583 (C=C), 1078, 1033 (C-O) cm^−^^1^. ^1^H-NMR (DMSO-*d6*, 400 MHz, ppm): δ 2.65 (s, 3H, CH_3_), 4.05 (s, 2H, CH_2_ of imidazolidine ring), 7.13–7.15 (d, 1H, Ar-H), 7.55–7.62 (m, 3H, Ar-H), 7.88 (s, 1H, Ar-H), 7.90 (s, 1H, Ar-H), 7.92–7.95 (dd, 1H, Ar-H), 8.27 (d, 1H, Ar-H), 12.25 (br.s, 1H, NH), 13.31 (s, 1H, OH). ^13^C-NMR (DMSO-*d6*, 100 MHz, ppm): δ 174.1 (C=S), 166.1 (C=O), 164.4 (C=N), 162.6 (C─N=N), 152.4 (C─O), 145.1 (Ph─N=N), 131.3, 129.9, 129.6, 126.6, 124.8, 122.8, 122.7, 119.8, 118.7 (C─aromatic), 34.1 (CH_2_ of imidazole ring), 14.8 (CH_3_). Anal. Calcd. for C_17_H_15_N_5_O_2_S (M.wt = 353): C, 57.79; H, 4.25; N, 19.83. Found: C, 57.51; H, 4.04; N, 19.68.

#### 3.2.5. Synthesis of 1-[2-(4-Chlorophenyl)-2-oxoethyl]-3-[1-(2-hydroxy-5-(phenyl diazenyl) phenyl) ethylidene) amino]-2-thioxoimidazolidin-4-one (**6**)

A stirred solution of compound **5** (3.5 g, 0.01 mol) in dimethylformamide (25 mL) was treated at 0 °C with triethyl amine (3 g, 0.03 mol), followed by a dropwise addition of 4-chlorophenyacyl bromide (2.3 g, 0.01 mol). The resulting reaction mixture was allowed to reflux and was followed by TLC analysis. After being refluxed for 12 h, the TLC analysis showed almost a complete reaction. The resultant reaction mixture was cooled to an ambient temperature and poured into ice-water. The obtained mixture was carefully neutralized with dilute HCl (2%) to pH 7, during which a solid precipitate formed. The resulting solid was filtered off, washed with water, dried, and recrystallized using ethanol as a solvent to afford compound **6** as orange crystals. R*_f_*: 0.59 (PE: ethylacetate 3:2, visualized with UV illumination). Yield: 68%, m.p. 180 °C. FT-IR (KBr) _γmax_: 3415 (br.OH), 1705, 1693 (C=O), 1631 (C=N), 1605, 1587 (C=C), 1061, 1032, (C-O) cm^−^^1^. ^1^H-NMR (DMSO-*d6*, 400 MHz, ppm): δ 2.45, 2.63 (s, 3H, CH3 of two stereo isomers), 4.32 (s, 2H, CH_2_ of imidazole ring), 5.38(s, 2H, COCH_2_N), 7.12–7.14 (d, 1H, Ar-H), 7.51–8.20 (m, 1H, Ar-H), 13.14 (s, 1H, OH). ^13^C-NMR (DMSO-*d6*, 100 MHz, ppm): δ 197.45, 191.36 (C=O of ketone for the two isomer), 172.2 (C=S), 167.5 (C=O), 162.6 (C=N), 162.6 (C─N=N), 152.4 (C─O), 145.2 (Ph─N=N), 139.7, 138.6, 135.9, 133.5, 131.4, 130.6, 130.6, 129.9, 129.7, 129.3, 126.9, 124.9, 122.8, 122.8, 119.8, 118.8 (C─aromatic of two isomer), 56.5, 49.5 (NCH_2_CO), 33.3, 27.2 (CH_2_ of imidazole ring), 19.0, 14.8 (CH_3_ of two isomer). Anal. Calcd. for C_25_H_20_N_5_ClO_3_S (M.wt = 505.5): C, 59.35; H, 3.96; N, 13.85. Found: C, 59.13.; H, 3.69; N, 13.53.

#### 3.2.6. Synthesis of Methyl-3{3-[1-(2-hydroxy-5-(phenyldiazenyl) phenyl) ethylidene) amino]-4-oxo-2-thioxoimidazolidin-1-yl} propanoate (**7**)

The entitled compound was synthesized following the procedure applied for the synthesis of compound **6**. Briefly, to a stirred solution of compound **5** (3.5 g, 0.01 mol) in dimethylformamide (30 mL) at 0 °C was added triethyl amine (3 g, 0.03 mol) and methyl acrylate (0.86 g, 0.01 mol). After the reaction mixture was refluxed for 14 h, the mixture was cooled to room temperature and placed into ice-water. The resulting mixture was subsequently neutralized with 2% HCl solution and the obtained solid was collected by filtration, washed with water, and dried. The resultant crude solid was finally recrystallized with ethanol to furnish compound **7** as yellow crystals. R*_f_*: 0.56 (PE: ethylacetate 3:2, visualized with UV illumination). Yield: 63%, m.p. 160 °C. FT-IR (KBr)_γmax_: 3421 (br.OH), 1746, 1691 (C=O), 1631(C=N), 1611, 1591(C=C), 1083, 1051(C-O) cm^−^^1^. ^1^H-NMR (DMSO-*d6*, 400 MHz, ppm): δ 2.64 (s, 3H, CH_3_), 2.73–2.76 (t, 2H, COCH_2_─), 3.62 (s, 3H, OCH_3_), 3.99–4.02 (t, 2H, N-CH_2_), 4.09 (s, 2H, CH_2_ of imidazole ring), 7.10–7.12 (d, 1H, Ar-H), 7.53–7.60 (m, 3H, Ar-H), 7.86–7.92 (m, 3H, Ar-H), 8.23 (br. s, 1H, Ar-H), 13.18 (s, 1H, OH). ^13^C-NMR (DMSO-*d6*, 100 MHz, ppm): δ 172.0 (C=S), 171.5 (C=O of ester), 167.3 (C=O), 163.0 (C=N), 62.6 (C─N=N), 152.4 (C-O), 145.1 (Ph─N=N), 131.3, 129.9, 129.8, 126.9, 124.8, 122.8, 122.7, 119.7, 118.7 (C-aromatic), 52.1 (OCH_3_), 33.3 (CH_2_ of imidazole ring), 31.5 (NCH_2_), 28.8 (CH_2_CO), 14.8 (CH_3_). Anal. Calcd. for C_21_H_21_N_5_O_4_S (M.wt = 439): C, 57.40; H, 4.78; N, 15.94. Found: C, 57.19; H, 4.51; N, 15.61.

#### 3.2.7. Synthesis of Methyl 3-{3 [1-(2-acetoxy)-5-(phenyldiazenyl)phenyl) ethylidene)amino)-4-oxo-2-thioxoimidazolidin-1-yl} Propanoate (**8**)

Compounds **7** (4.4 g, 0.01 mol) was carefully treated with acetic anhydride (30 mL). After being stirred under reflux for 16 h, the TLC analysis showed almost a complete reaction. The reaction mixture was subsequently cooled to room temperature and then poured into pre-cooled water. The obtained mixture was allowed to stand at 4 °C for an additional 16 h, before it was filtered. The resultant solid was washed with water, dried, and finally recrystallized using ethanol to provide compound **8** as pale orange crystals. R*_f_*: 0.42 (PE: ethylacetate 4:1, visualized with UV illumination). Yield: 61%, m.p. 130 °C. FT-IR (KBr)_γ max_: 1753–1749 (C=O of ester), 1641 (C=O), 1625 (C=N), 1610, 1591 (C=C), 1087, 1038 (C-O) cm^−^^1^. ^1^H-NMR (DMSO-*d6*, 400 MHz, ppm): δ 1.71 (S, 3H, CH_3_), 2.65 (s, 3H, CH_3_), 2.74–2.77 (t, 2H, CH_2_O), 3.62 (s, 3H, OCH_3)_, 4.01–4.04 (t, 2H, NCH_2_), 4.09 (s, 2H, CH_2_ of imidazole ring), 7.08–8.24 (m, 8H, Ar-H). ^13^C-NMR (DMSO-*d6*, 100 MHz, ppm): δ 172.1 (C=S), 171.5, 167.4 (C=O), 162.8 (C=N), 162.6 (C─N=N), 152.5 (C-O), 145.3 (Ph─N=N), 131.1, 129.8, 129.5, 127.0, 124.9, 122.7, 121.7, 119.0 (C─Aromatic), 52.1 (OCH_3_), 33.2, (CH_2_ of imidazole ring), 31.5 (NCH_2_), 30.6 (CH_2_ CO), 15.4 (CH_3_). Anal. Calcd. for C_23_H_23_N_5_O_5_S (M.wt = 481): C, 57.38; H, 4.78; N, 14.55. Found: C, 57.13; H, 4.54; N, 14.33.

### 3.3. Cell Line and Culture 

RAW264.7 cells, monocyte/macrophage obtained from murine (product no. 91062702). Dulbecco’s modified Eagle’s minimum essential medium (DMEM) (product no. D5030) was used to proliferate the cells with 5% fetal bovine serum (product no. F0926) in a cool and dry environment of 5% CO_2_ at 37 °C. Monocytes and macrophages are significant elements in the immune system since they are key inflammatory cells involved in the induction of inflammatory reactions through infection. Their activation results in many inflammatory diseases by releasing several proinflammatory cytokines and mediators, and generating ROS, all of which contribute considerably to the genesis of inflammatory disorders. As a result, modulating macrophage-mediated inflammatory reactions is critical for establishing a unique therapy strategy for chronic inflammatory diseases. Different stimuli, including LPS (the main element of the outer membrane derived from Gram-negative bacteria) (product no. L4391), are considered the main initial factors of the inflammatory reaction by exciting macrophages through multiple signaling pathways. By introducing LPS (5 μg/mL) to the murine macrophage cell line RAW264.7, endotoxin-induced inflammation was studied in vitro using LPS-treated macrophages [93]. 

### 3.4. Assessment of Cytotoxicity against LPS-Activated RAW264.7 Cell Line Using MTT Assay 

3-(4,5-dimethylthiazol-2-yl)-2,5 diphenyltetrazolium bromide (MTT), a hydrogen acceptor, is usually used to assess the viability of cells by observing the yellow MTT converted to its purple product and MTT formazan, which differs among cell lines. The quantity of formazan is directly proportional to the amount of alive cells and is determined by documenting the variations in absorbance at wavelength 570 nm using a plate reading spectrophotometer. As the alive cells transfer MTT to the colored response result purple colored formazan, the dead cells cannot transfer MTT into formazan. According to [94], the 96 well plate that contained the tissue culture was loaded with 1 × 10^5^ cells/mL (100 μL/well) and kept at 37 °C for a day to form a full monolayer sheet. When the adherent layer of cells formed, the growth medium was transferred from 96 well micro titer plates; this cell monolayer was washed two times with wash media, after that, the tested samples with two-fold dilutions were put in RPMI medium with 2% serum (cultured medium). Then, 0.1 mL of each dilution was examined in separate wells. Plates were maintained at 37 °C. MTT mixture was carried out (5 mg/mL in PBS) (product number CT02). Following this, 20 uL MTT solution was added to each well and then the plate was shaken. The plate was then incubated (37 °C with CO_2_ 5%) for 4 h to ensure the MTT was processed. The media was thrown away and the metabolic product (formazan) of the MTT was soaked in 200 uL DMSO and mixed well. The optical density was documented at a wavelength of 560 nm which was directly related to the number of cells. The cytotoxicity of 1,3 disubstituted-2-thioxoimidazolidin-4-one derivatives (**3**–**8**) at 5 different concentrations (1000, 250, 63, 16, 4 μg/mL) were examined against the RAW264.7 cell line that activated with LPS (5 μg/mL) to enhance inflammation reactions using MTT assay. Celecoxib (product no. SML3031), a COX-2 inhibitor and anti-inflammatory drug, was utilized as a reference compound. The outcomes were recorded as a % of viable cells in comparison to the celecoxib subjected cells. 

### 3.5. Assessment of the Anti-Inflammatory Activity against LPS-Activated RAW264.7 Cell Line by Estimating NO Production

To evaluate the impacts of 1,3 disubstituted-2-thioxoimidazolidin-4-one derivatives (**3**–**8**) on the NO production, we assessed the amounts of nitrite, an indicator of NO formation, produced by LPS when added to RAW264.7 cells that induced inflammatory response and increased the manufacture of NO. The RAW264.7 cells were placed on a 96-well plate and were pre-treated with 1,3 disubstituted-2-thioxoimidazolidin-4-one derivatives (**3**–**8**) for 1 hour after overnight culture (1 × 10^5^ cells/well, 500 μL medium/well), and then treated with LPS (5 μg/mL) for 1 day; the culture supernatant was then gathered then utilized to estimate NO production by Griess reaction using a colorimetric kit (product no. MAK454). Celecoxib, a COX-2 inhibitor and anti-inflammatory drug, was utilized as a reference compound [95]. 

### 3.6. Assessment of the Expression of IL-1β by Western Blot Analysis 

The anti-inflammatory impact of compound **7** (10 and 50 μg/mL) on the expression of IL-1 in RAW264.7 cells stimulated with LPS (5 μg/mL) was evaluated by Western blot analysis using the manufacturer’s suggested normalization to β-actin protein [96]. The cells were treated with compound **7** with either one of the 2 doses (10 and 50 μg/mL) or DMSO (control), then added cold lysis buffer (250 μL) including Tris-buffered saline (0.1%, pH 7.4), and immediately increased with 1:400 protease and phosphatase inhibitors combination (product no. PPC1010). The cells were then defrosted, gathered and centrifuged at 12,000 rpm/10 min/4 °C. After that 30 μg of protein was loaded into SDS-polyacrylamide gel to separate by an electrophoresis unit (Cleaver Scientific Ltd, UK). Afterward, the protein moved to polyvinylidene fluoride membranes (product no. 3010040001) for 35 min, then was blocked in 5% bovine serum albumin (BSA) (product no. A2153) in Tris-buffered saline, with 0.05% Tween ® (TBST), (product no. T9039). The membranes were blocked overnight at 4 °C with the primary antibody diluted in BSA (1:1000) and washed four times with 1 TBST buffer, followed by the secondary antibody conjugated with horseradish peroxidase diluted in TBST buffer (1:2000) for one hour at RT. Western Lightning enhanced chemiluminescence (ECL) chemicals (product no. GERPN2209) were used to identify the signals, which were photographed by a ChemiDoc imager (Bio-Rad, Hercules, CA, USA). Normalization to -actin was used to determine band intensities.

### 3.7. Assessment of the Expression of IL-6 and TNF-α Cytokines by Real-Time PCR

After evaluating the previous assessments on the 6 derivatives of 1,3 disubstituted-2-thioxoimidazolidin-4-one, we found that derivative 7 (compound **7**) had the most potent anti-inflammatory effect among the other 6 compounds, so we selected compound **7** due to its effect on inflammatory cytokines in vitro. LPS-activated RAW264.7 cells were treated by compound **7** (50 μg/mL) for 2 days at 37 °C before being collected for real-time PCR measurement of IL-6 and TNF-α expression in comparison to control cells without treatment, and Celecoxib (50 μg/mL) was utilized as a reference compound. Extracted total RNA was made by RNeasy Mini Kit (Qiagen, Hilden, Germany), then cDNA synthesis kit (product no. 11117831001) was used to make cDNA from 1 μg RNA. qRT-PCR was performed with 10 ng cDNA. The cycling conditions were initial heating stage 5 min at 95 °C, then 45 cycles of denaturation phase at 95 °C 10 s, annealing phase at 60 °C 30 s, and extension phase at 72 °C 1 min using iScript^TM^ One-Step RT-PCR Kit with SYBR^®^ Green (Bio-Rad, Hercules, CA, USA). Finally, the expression of relative gene was carried out using 2^−ΔΔct^ equation and normalized to β-actin gene [66,97]. Primers of IL-6 were F 5′-AGACAGCCACTCACCTCTTCAG-3′, R 5′-TTCTGCCAGTGCCTCTTTGCTG-3′; primers of TNF-α were F 5′-CTCTTCTGCCTGCTGCACTTTG-3′, R 5′-ATGGGCTACAGGCTTGTCACTC-3′; primers of β-actin were F 5′-GCACCACACCTTCTACAATG-3′, R 5′-TGCTTGCTGATCCACATCTG-3′.

### 3.8. In Silico Molecular Docking Analysis

To explore whether the observed anti-inflammatory activity of this class of compounds could be a result of their ability to target Cyclooxygenases enzymes, extensive molecular docking studies were performed using MOE software. Computational analysis affirmed the ability to assess the binding affinity of small molecules (ligand) toward the active pocket of the targeted protein (receptor) and offer valuable information to explain the mode of action of pharmacological compounds [97,98,99,100,101,102]. To examine the binding mode of mono N3-substituted and N1, N3-disubstituted-2-thohydantoins, compounds **5** and **7** were selected to explore their binding affinity toward the active pocket of COX1 and COX-2 enzymes. The 2D-structure of compounds **5** and **7** was obtained by utilizing Chem. Draw software. The X-ray structures of COX-1 (PDB code: *3KK6*) and COX-2 (PDB code: *3LN1*) were acquired from the protein databank RCSB (http://www.rcsb.org/, accessed on 1 June 2022) [103,104]. The preparation of COX1 and 2 proteins, 3D-structures of compounds **5** and **7**, and the molecular modelling study were achieved utilizing the MOE program as previously reported [97,98,99,100,101,102]. The docking protocol was adjusted to Triangle Matcher placement and the scoring function to London dG. The applied protocol was examined for validity by evaluating the binding affinity and mode of interactions of the original co-crystallized ligand compared to the reported data. The obtained data were assessed, and the binding modes with high binding affinity were chosen to estimate the score and binding energy.

### 3.9. Statistical Analysis

All tests were carried out three times. The records are reported in the form of the mean standard deviation. One-way ANOVA was used to evaluate the records, followed by the Bonferroni post hoc test. The *p* value was used to establish the relevance of the data; *p* > 0.05 is regarded non-significant, whereas *p* < 0.05 is considered significant. For statistical assessment, we used GraphPad prism software Incorporation (San Diego, CA, USA, 2007) [101,102].

## 4. Conclusions

In the presented study, we described the design and synthesis of a novel set of 1,3-disubstituted-2-thioxoimidazolidin-4-one derivatives. The anti-inflammatory activity of synthesized compounds was examined in an LPS-induced murine leukemia cell line (RAW264.7) by evaluating their cytotoxicity activity and their ability to diminish the NO production. Our investigations revealed that this class of compounds possesses considerable anti-inflammatory activity. Compound **5** and **7**, among the synthesized compounds, demonstrated the most potent anti-inflammatory activity compared to the celecoxib drug. These compounds showed significant cytotoxic activity toward the LPS-induced murine leukemia cell line and a substantial ability to reduce NO production. Exploration of the mode of anti-inflammatory action of this class of compounds revealed that compound **7**, a representative compound and the most potent analogue, displays the ability to significantly reduce the expression of the anti-inflammatory cytokines (IL-6, TNF-α, and IL-1β). Furthermore, a detailed in silico molecular docking study indicated that compound **7** exhibits considerable binding affinity toward the COX-2 binding cavity, suggesting that compound **7** might possess inhibitory activity towards the COX-2 enzyme. Together, this study showed, for the first time, that the 1,3-disubstituted-2-thiohydantoins scaffold could be considered for the development of novel and effective anti-inflammatory agents. 

## Figures and Tables

**Table 1:** Binding affinity scores and interactions of compounds **5** and **7** into the binding cavity of COX-1 (PDB: *3kk6*) and COX-2 (PDB: *3ln1*) proteins.

Protein (PDB Code)	Compound	Hydrophilic Interactions	Distance (A)	Hydrophobic Interactions	S (kcal/mol)
COX-1 (*3kk6*)	**5**	Ser530	2.78	Val116, Val349, Leu352, Trp387, Ile517, Phe318, Ala527, Met522, Ile523, Leu531	−8.36
	**7**	Ser353 Arg120	3.23 2.94	Leu93, Val116, Leu359, Ile345, Phe381, Trp387, Leu384, Leu352, Val349, Lez534, Leu531, Ala527, Met522, Phe518, Ile523	−9.54
COX-2 (*3ln1*)	**5**	Ser516 Ser516 Val509	2.96 3.34 3.59	Val335, Leu338, Phe367, Trp373, Ala502, Ile503, Phe504, Met508, Ala513, Leu517	−9.96
	**7**	Ser516 Ser516 Val509 Arg106	1.97 2.75 3.88 3.46	Val102, Leu345, Val335, Leu370, Trp373, Leu338, Phe367, Met508, Ala513, Ala502, Ile503, Phe504, Leu517	−11.17

## Data Availability

Not applicable.

## Supplementary Materials

The following supporting information can be downloaded at: https://www.mdpi.com/article/10.3390/molecules27196271/s1, Figure S1. ^1^H-NMR and ^13^C-NMR spectra of compound **3** in DMSO-*d_6_*; Figure S2. ^1^H-NMR and ^13^C-NMR spectra of compound **4** in DMSO-*d_6_*; Figure S3. ^1^H-NMR and ^13^C-NMR spectra of compound **5** in DMSO-*d_6_*; Figure S4. ^1^H-NMR and ^13^C-NMR spectra of compound **6** in DMSO-*d_6_*; Figure S5. ^1^H-NMR and ^13^C-NMR spectra of compound **7** in DMSO-*d_6_*; Figure S6. ^1^H-NMR and ^13^C-NMR spectra of compound **8** in DMSO-*d_6_*; Figure S7. Western plot analysis of the in vitro anti-inflammatory effect of compound **7** against LPS- activated RAW264.7 cell line on IL-1β expression; Table S1: *In vitro* cytotoxicity of 1,3 disubstituted-2-thioxoimidazolidin-4-one derivatives against LPS-induced RAW264.7; Table S2: Effect of compound **7** on IL-6 and TNF-α expression in RAW264.7 by RT-PCR; Table S3: *In vitro* effect of compound **7** on Western blot analysis of IL-1β in RAW264.7 cells.

## Sample Availability

Samples of the compounds **2**–**8** are available from the authors.

## References

[1] Barton G.M. (2008). A calculated response: Control of inflammation by the innate immune system. J. Clin. Investig..

[2] Ahmad R., Ahsan H. (2022). Dual Autoimmune diseases: Rheumatoid arthritis with systemic lupus erythematosus and Type 1 diabetes mellitus with multiple sclerosis. Rheumatol. Autoimmun..

[3] El-Sharief M.A.M.S., Abbas S.Y., El-Sharief A.M.S., Sabry N.M., Moussa Z., El-Messery S.M., Elsheakh A.R., Hassan G.S., El Sayed M.T. (2019). 5-Thioxoimidazolidine-2-one derivatives: Synthesis, anti-inflammatory activity, analgesic activity, COX inhibition assay and molecular modelling study. Bioorg. Chem..

[4] da Silva Guerra A.S.H., do Nascimento Malta D.J., Morais Laranjeira L.P., Souza Maia M.B., Cavalcanti Colaço N., do Carmo Alves de Lima M., Galdino S.L., da Rocha Pitta I., Gonçalves-Silva T. (2011). Anti-inflammatory and antinociceptive activities of indole—Imidazolidine derivatives. Int. Immunopharmacol..

[5] Bian M., Ma Q., Wu Y., Du H., Guo-hua G. (2021). Small molecule compounds with good anti-inflammatory activity reported in the literature from 01/2009 to 05/2021: A review. J. Enzym. Inhib. Med. Chem..

[6] Wojdasiewicz P., Poniatowski Ł.A., Szukiewicz D. (2014). The role of inflammatory and anti-inflammatory cytokines in the pathogenesis of osteoarthritis. Mediat. Inflamm..

[7] Idriss H.T., Naismith J.H. (2000). TNF alpha and the TNF receptor superfamily: Structure-function relationship(s). Microsc. Res. Tech..

[8] Jang D., Lee A.-H., Shin H.-Y., Song H.-R., Park J.-H., Kang T.-B., Lee S.-R., Yang S.-H. (2021). The role of tumor necrosis factor alpha (TNF-α) in autoimmune disease and current TNF-α inhibitors in therapeutics. Int. J. Mol. Sci..

[9] Lopez-Castejon G., Brough D. (2011). Understanding the mechanism of IL-1β secretion. Cytokine Growth Factor Rev..

[10] Velazquez-Salinas L., Verdugo-Rodriguez A., Rodriguez L.L., Borca M.V. (2019). The role of interleukin 6 during viral infections. Front. Microbiol..

[11] Rose-John S., Winthrop K., Calabrese L. (2017). The role of IL-6 in host defence against infections: Immunobiology and clinical implications. Nat. Rev. Rheumatol..

[12] Attiq A., Jalil J., Husain K., Ahmad W. (2018). Raging the war against inflammation with natural products. Front. Pharmacol..

[13] Bindu S., Mazumder S., Bandyopadhyay U. (2020). Non-steroidal anti-inflammatory drugs (NSAIDs) and organ damage: A current perspective. Biochem. Pharmacol..

[14] Gunaydin C., Bilge S.S. (2018). Effects of nonsteroidal anti-inflammatory drugs at the molecular level. Eurasian J. Med..

[15] Zarghi A., Arfaei S. (2011). Selective COX-2 Inhibitors: A review of their structure-activity relationships. Iran. J. Pharm. Res. IJPR.

[16] Muccioli G.G., Fazio N., Scriba G.K.E., Poppitz W., Cannata F., Poupaert J.H., Wouters J., Lambert D.M. (2006). Substituted 2-Thioxoimidazolidin-4-ones and imidazolidine-2,4-diones as fatty acid amide hydrolase inhibitors templates. J. Med. Chem..

[17] Kim H.R., Lee H.J., Choi Y.J., Park Y.J., Woo Y., Kim S.J., Park M.H., Lee H.W., Chun P., Chung H.Y. (2014). Benzylidene-linked thiohydantoin derivatives as inhibitors of tyrosinase and melanogenesis: Importance of the β-Phenyl-α,β-unsaturated carbonyl functionality. MedChemComm.

[18] Marton J., Enisz J., Hosztafi S., Timar T. (1993). Preparation and fungicidal activity of 5-substituted hydantoins and their 2-thio analogs. J. Agric. Food Chem..

[19] Han J., Dong H., Xu Z., Lei J., Wang M. (2013). Facile synthesis of 5-arylidene thiohydantoin by sequential sulfonylation/desulfination reaction. Int. J. Mol. Sci..

[20] Tejchman W., Orwat B., Korona-Głowniak I., Barbasz A., Kownacki I., Latacz G., Handzlik J., Żesławska E., Malm A. (2019). Highly efficient microwave synthesis of rhodanine and 2-thiohydantoin derivatives and determination of relationships between their chemical structures and antibacterial activity. RSC Adv..

[21] Tejchman W., Korona-Glowniak I., Malm A., Zylewski M., Suder P. (2017). Antibacterial properties of 5-substituted derivatives of rhodanine-3-carboxyalkyl acids. Med. Chem. Res..

[22] Camargo P.G., da Silva Bortoleti B.T., Fabris M., Gonçalves M.D., Tomiotto-Pellissier F., Costa I.N., Conchon-Costa I., da Silva Lima C.H., Pavanelli W.R., de Lima Ferreira Bispo M. (2022). Thiohydantoins as anti-leishmanial agents: N vitro biological evaluation and multi-target investigation by molecular docking studies. J. Biomol. Struct. Dyn..

[23] Buchynskyy A., Gillespie J.R., Herbst Z.M., Ranade R.M., Buckner F.S., Gelb M.H. (2017). 1-Benzyl-3-aryl-2-thiohydantoin derivatives as new anti-*Trypanosoma brucei* agents: SAR and in vivo efficacy. ACS Med. Chem. Lett..

[24] Wu F., Jiang H., Zheng B., Kogiso M., Yao Y., Zhou C., Li X.-N., Song Y. (2015). Inhibition of cancer-associated mutant isocitrate dehydrogenases by 2-thiohydantoin compounds. J. Med. Chem..

[25] Cho S., Kim S.-H., Shin D. (2019). Recent Applications of Hydantoin and thiohydantoin in medicinal chemistry. Eur. J. Med. Chem..

[26] Tran C., Ouk S., Clegg N.J., Chen Y., Watson P.A., Arora V., Wongvipat J., Smith-Jones P.M., Yoo D., Kwon A. (2009). Development of a second-generation antiandrogen for treatment of advanced prostate cancer. Science.

[27] Lee T.H., Khan Z., Kim S.Y., Lee K.R. (2019). Thiohydantoin and hydantoin derivatives from the roots of armoracia rusticana and their neurotrophic and anti-neuroinflammatory activities. J. Nat. Prod..

[28] Haslak Z.P., Cinar S.A., Ozbek S.S., Monard G., Dogan I., Aviyente V. (2020). Elucidation of the atroposelectivity in the synthesis of axially chiral thiohydantoin derivatives. Org. Biomol. Chem..

[29] Králová P., Maloň M., Koshino H., Soural M. (2018). Convenient synthesis of thiohydantoins, imidazole-2-thiones and imidazo [2,1-b]Thiazol-4-Iums from polymer-supported α-acylamino ketones. Molecules.

[30] Metwally M.A., Abdel-Latif E. (2012). Thiohydantoins: Synthetic strategies and chemical reactions. J. Sulfur Chem..

[31] Muccioli G.G., Poupaert J.H., Wouters J., Norberg B., Poppitz W., Scriba G.K.E., Lambert D.M. (2003). A Rapid and efficient microwave-assisted synthesis of hydantoins and thiohydantoins. Tetrahedron.

[32] Elokdah H., Sulkowski T.S., Abou-Gharbia M., Butera J.A., Chai S.-Y., McFarlane G.R., McKean M.-L., Babiak J.L., Adelman S.J., Quinet E.M. (2004). Design, synthesis, and biological evaluation of thio-containing compounds with serum HDL-cholesterol-elevating properties. J. Med. Chem..

[33] Kokotos C.G., Limnios D., Triggidou D., Trifonidou M., Kokotos G. (2011). Novel pyrrolidine-thiohydantoins/thioxotetrahydropyrimidinones as highly effective catalysts for the asymmetric michael addition. Org. Biomol. Chem..

[34] Montagne C., Shipman M. (2006). Modified bucherer-bergs reaction for the one-pot synthesis of 5, 5′-disubstituted hydantoins from nitriles and organometallic reagents. Synlett.

[35] Wang Z.D., Sheikh S.O., Zhang Y. (2006). A Simple synthesis of 2-thiohydantoins. Molecules.

[36] Majumdar P., Bathula C., Basu S.M., Das S.K., Agarwal R., Hati S., Singh A., Sen S., Das B.B. (2015). Design, synthesis and evaluation of thiohydantoin derivatives as potent topoisomerase i (top1) inhibitors with anticancer activity. Eur. J. Med. Chem..

[37] Gauthier M.P., Michaux C., Rolin S., Vastersaegher C., de Leval X., Julémont F., Pochet L., Masereel B. (2006). Synthesis, molecular modelling and enzymatic evaluation of (±)3,5-diphenyl-2-thioxoimidazolidin-4-ones as new potential cyclooxygenase inhibitors. Bioorg. Med. Chem..

[38] Brandao S.S.F., Andrade A.M.C., Pereira D.Τ.M., Filho J.M.B., Lima M.C.A., Galdino S.L., Pitta I.R., Barbe J. (2004). A novel way of synthesis of l,3,5-trisubstituted-2-thioxoimidazolidinones. Heterocycl. Commun..

[39] Abdellatif K.R.A., Fadaly W.A.A., Mostafa Y.A., Zaher D.M., Omar H.A. (2019). Thiohydantoin derivatives incorporating a pyrazole core: Design, synthesis and biological evaluation as dual inhibitors of topoisomerase-I and cycloxygenase-2 with anti-cancer and anti-inflammatory activities. Bioorg. Chem..

[40] Park H.S., Choi H.J., Shin H.S., Park M.S., Lee S.K. (2007). Synthesis and characterization of novel hydantoins as potential COX-2 inhibitors: 1,5-diarylhydantoins. Bull. Korean Chem. Soc..

[41] Gediya L.K., Njar V.C. (2009). Promise and challenges in drug discovery and development of hybrid anticancer drugs. Expert Opin. Drug Discov..

[42] Rialdi A., Campisi L., Zhao N., Lagda A.C., Pietzsch C., Ho J.S.Y., Martinez-Gil L., Fenouil R., Chen X., Edwards M. (2016). Topoisomerase 1 inhibition suppresses inflammatory genes and protects from death by inflammation. Science.

[43] Viegas-Junior C., Danuello A., da Silva Bolzani V., Barreiro E.J., Fraga C.A.M. (2007). Molecular hybridization: A useful tool in the design of new drug prototypes. Curr. Med. Chem..

[44] Fershtat L.L., Makhova N.N. (2017). Molecular hybridization tools in the development of furoxan-based no-donor prodrugs. ChemMedChem.

[45] Sztanke K., Maziarka A., Osinka A., Sztanke M. (2013). An insight into synthetic schiff bases revealing antiproliferative activities in vitro. Bioorg. Med. Chem..

[46] Kajal A., Bala S., Kamboj S., Sharma N., Saini V. (2013). Schiff bases: A versatile pharmacophore. J. Catal..

[47] Chigurupati S., Selvaraj M., Mani V., Mohammad J.I., Selvarajan K.K., Akhtar S.S., Marikannan M., Raj S., Teh L.K., Salleh M.Z. (2018). Synthesis of azomethines derived from cinnamaldehyde and vanillin: In vitro aetylcholinesterase inhibitory, antioxidant and insilico molecular docking studies. Med. Chem. Res..

[48] Ali Channar P., Bano S., Hassan S., Perveen F., Saeed A., Ali Mahesar P., Ali Khan I., Iqbal J. (2022). Appraisal of novel azomethine—Thioxoimidazolidinone conjugates as ecto-5′-nucleotidase inhibitors: Synthesis and molecular docking studies. RSC Adv..

[49] Saied E.M., Arenz C. (2021). Stereoselective synthesis of novel sphingoid bases utilized for exploring the secrets of sphinx. Int. J. Mol. Sci..

[50] Saied E.M., Diederich S., Arenz C. (2014). Facile synthesis of the CERT inhibitor HPA-12 and some novel derivatives. Chem. Asian J..

[51] Saied E.M., Banhart S., Bürkle S.E., Heuer D., Arenz C. (2015). A series of ceramide analogs modified at the 1-position with potent activity against the intracellular growth of chlamydia trachomatis. Future Med. Chem..

[52] Abdel-Wahab B.A., Abd El-Kareem H.F., Alzamami A., Fahmy C.A., Elesawy B.H., Mostafa Mahmoud M., Ghareeb A., El Askary A., Abo Nahas H.H., GM Attallah N. (2022). Novel exopolysaccharide from marine bacillus subtilis with broad potential biological activities: Insights into antioxidant, anti-inflammatory, cytotoxicity, and anti-alzheimer activity. Metabolites.

[53] Banhart S., Saied E.M., Martini A., Koch S., Aeberhard L., Madela K., Arenz C., Heuer D. (2014). Improved plaque assay identifies a novel anti-chlamydia ceramide derivative with altered intracellular localization. Antimicrob. Agents Chemother..

[54] Salem M.G., El-Maaty D.M.A., El-Deen Y.I.M., Elesawy B.H., Askary A.E., Saleh A., Saied E.M., Behery M.E. (2022). Novel 1,3-thiazole analogues with potent activity against breast cancer: A design, synthesis, in vitro, and in silico study. Molecules.

[55] El Azab I.H., Saied E.M., Osman A.A., Mehana A.E., Saad H.A., Elkanzi N.A. (2021). Novel N-bridged pyrazole-1-carbothioamides with potential antiproliferative activity: Design, synthesis, in vitro and in silico studies. Future Med. Chem..

[56] Gaber A., Alsanie W.F., Kumar D.N., Refat M.S., Saied E.M. (2020). Novel papaverine metal complexes with potential anticancer activities. Molecules.

[57] Samaha D., Hamdo H.H., Cong X., Schumacher F., Banhart S., Aglar Ö., Möller H.M., Heuer D., Kleuser B., Saied E.M. (2020). Liposomal FRET assay identifies potent drug-like inhibitors of the ceramide transport protein (CERT). Chem. Eur. J..

[58] Refat M.S., Ibrahim H.K., Sowellim S.Z.A., Soliman M.H., Saeed E.M. (2009). Spectroscopic and thermal studies of Mn (II), Fe (III), Cr (III) and Zn (II) complexes derived from the ligand resulted by the reaction between 4-acetyl pyridine and thiosemicarbazide. J. Inorg. Organomet. Polym. Mater..

[59] Csonka F.A., Nicolet B.H. (1932). The preparation of optically active thiohydantoins and the racemization of amino acids as their azlactones. J. Biol. Chem..

[60] Inglis A.S., Duncan M.W., Adams P., Tseng A. (1992). Formation of proline thiohydantoin with ammonium thiocyanate: Progress towards a viable C-terminal amino-acid-sequencing procedure. J. Biochem. Biophys. Methods.

[61] Rossol M., Heine H., Meusch U., Quandt D., Klein C., Sweet M.J., Hauschildt S. (2011). LPS-induced cytokine production in human monocytes and macrophages. Crit. Rev. Immunol..

[62] Yücel G., Zhao Z., El-Battrawy I., Lan H., Lang S., Li X., Buljubasic F., Zimmermann W.-H., Cyganek L., Utikal J. (2017). Lipopolysaccharides induced inflammatory responses and electrophysiological dysfunctions in human-induced pluripotent stem cell derived cardiomyocytes. Sci. Rep..

[63] Page M.J., Kell D.B., Pretorius E. (2022). The role of lipopolysaccharide-induced cell signalling in chronic inflammation. Chronic Stress.

[64] Battistone M.J., Sawitzke A.D. (2016). Clinical medicine insights: Therapeutics celecoxib in the treatment of osteoarthritis. Clin. Med. Insights Ther..

[65] Elhady H.A., El-Sayed R., Al-nathali H.S. (2018). Design, synthesis and evaluation of anticancer activity of novel 2-thioxoimidazolidin-4-one derivatives bearing pyrazole, triazole and benzoxazole moieties. Chem. Cent. J..

[66] Nafie M.S., Khodair A.I., Hassan H.A.Y., El-Fadeal N.M.A., Bogari H.A., Elhady S.S., Ahmed S.A. (2021). Evaluation of 2-thioxoimadazolidin-4-one derivatives as potent anti-cancer agents through apoptosis induction and antioxidant activation: In vitro and in vivo approaches. Molecules.

[67] Han S., Gao H., Chen S., Wang Q., Li X., Du L.-J., Li J., Luo Y.-Y., Li J.-X., Zhao L.-C. (2019). Procyanidin A1 alleviates inflammatory response induced by LPS through NF-ΚB, MAPK, and Nrf2/HO-1 pathways in RAW264.7 cells. Sci. Rep..

[68] Xue B., Wu Y., Yin Z., Zhang H., Sun S., Yi T., Luo L. (2005). Regulation of lipopolysaccharide-induced inflammatory response by glutathione S-transferase P1 in RAW264.7 cells. FEBS Lett..

[69] Monga S., Fares B., Yashaev R., Melamed D., Kahana M., Fares F., Weizman A., Gavish M. (2022). The effect of natural-based formulation (NBF) on the response of RAW264.7 macrophages to LPS as an in vitro model of inflammation. J. Fungi.

[70] Li H., Zhang Q., Jin X., Zou X., Wang Y., Hao D., Fu F., Jiao W., Zhang C., Lin H. (2018). Dysifragilone a inhibits LPS-induced RAW264.7 macrophage activation by blocking the P38 MAPK signaling pathway. Mol. Med. Rep..

[71] Tanaka T., Narazaki M., Kishimoto T. (2014). IL-6 in inflammation, immunity, and disease. Cold Spring Harb. Perspect. Biol..

[72] Luo Y., Zheng S.G. (2016). Hall of fame among pro-inflammatory cytokines: Interleukin-6 gene and its transcriptional regulation mechanisms. Front. Immunol..

[73] Khalifa S.A.M., Shedid E.S., Saied E.M., Jassbi A.R., Jamebozorgi F.H., Rateb M.E., Du M., Abdel-Daim M.M., Kai G.-Y., Al-Hammady M.A.M. (2021). Cyanobacteria—From the Oceans to the Potential Biotechnological and Biomedical Applications. Marine Drugs.

[74] Vigil S.V.G., de Liz R., Medeiros Y.S., Fröde T.S. (2008). Efficacy of tacrolimus in inhibiting inflammation caused by carrageenan in a murine model of air pouch. Transpl. Immunol..

[75] Yu W., Guo Z., Orth P., Madison V., Chen L., Dai C., Feltz R.J., Girijavallabhan V.M., Kim S.H., Kozlowski J.A. (2010). Discovery and SAR of hydantoin TACE inhibitors. Bioorg. Med. Chem. Lett..

[76] Pinzi L., Rastelli G. (2019). Molecular docking: Shifting paradigms in drug discovery. Int. J. Mol. Sci..

[77] Salmaso V., Moro S. (2018). Bridging molecular docking to molecular dynamics in exploring ligand-protein recognition process: An overview. Front. Pharmacol..

[78] Sliwoski G., Kothiwale S., Meiler J., Lowe E.W. (2014). Computational methods in drug discovery. Pharmacol. Rev..

[79] Torres P.H.M., Sodero A.C.R., Jofily P., Silva F.P. (2019). Key topics in molecular docking for drug design. Int. J. Mol. Sci..

[80] Wang Z., Sun H., Yao X., Li D., Xu L., Li Y., Tian S., Hou T. (2016). Comprehensive evaluation of ten docking programs on a diverse set of protein-ligand complexes: The prediction accuracy of sampling power and scoring power. Phys. Chem. Chem. Phys..

[81] Lee S.H., Soyoola E., Chanmugam P., Hart S., Sun W., Zhong H., Liou S., Simmons D., Hwang D. (1992). Selective expression of mitogen-inducible cyclooxygenase in macrophages stimulated with lipopolysaccharide. J. Biol. Chem..

[82] Maier J.A., Hla T., Maciag T. (1990). Cyclooxygenase is an immediate-early gene induced by interleukin-1 in human endothelial cells. J. Biol. Chem..

[83] Sirois J., Richards J.S. (1992). Purification and characterization of a novel, distinct isoform of prostaglandin endoperoxide synthase induced by human chorionic gonadotropin in granulosa cells of rat preovulatory follicles. J. Biol. Chem..

[84] Kawaguchi H., Pilbeam C.C., Gronowicz G., Abreu C., Fletcher B.S., Herschman H.R., Raisz L.G., Hurley M.M. (1995). Transcriptional induction of prostaglandin G/H synthase-2 by basic fibroblast growth factor. J. Clin. Investig..

[85] Xie W., Herschman H.R. (1996). Transcriptional regulation of prostaglandin synthase 2 gene expression by platelet-derived growth factor and serum. J. Biol. Chem..

[86] Ghlichloo I., Gerriets V. (2022). Nonsteroidal Anti-Inflammatory Drugs (NSAIDs).

[87] Brune K., Patrignani P. (2015). New insights into the use of currently available non-steroidal anti-inflammatory drugs. J. Pain Res..

[88] Ahmadi M., Bekeschus S., Weltmann K.-D., von Woedtke T., Wende K. (2022). Non-steroidal anti-inflammatory drugs: Recent advances in the use of synthetic COX-2 inhibitors. RSC Med. Chem..

[89] Shiff S.J., Shivaprasad P., Santini D.L. (2003). Cyclooxygenase inhibitors: Drugs for cancer prevention. Curr. Opin. Pharmacol..

[90] Masferrer J.L., Leahy K.M., Koki A.T., Zweifel B.S., Settle S.L., Woerner B.M., Edwards D.A., Flickinger A.G., Moore R.J., Seibert K. (2000). Antiangiogenic and antitumor activities of cyclooxygenase-2 inhibitors. Cancer Res..

[91] Hoozemans J.J.M., Veerhuis R., Rozemuller A.J.M., Eikelenboom P. (2003). Non-steroidal anti-inflammatory drugs and cyclooxygenase in alzheimer’s disease. Curr. Drug Targets.

[92] Teismann P., Tieu K., Choi D.-K., Wu D.-C., Naini A., Hunot S., Vila M., Jackson-Lewis V., Przedborski S. (2003). Cyclooxygenase-2 is instrumental in parkinson’s disease neurodegeneration. Proc. Natl. Acad. Sci. USA.

[93] Zong Y., Sun L., Liu B., Deng Y.-S., Zhan D., Chen Y.-L., He Y., Liu J., Zhang Z.-J., Sun J. (2012). Resveratrol inhibits LPS-induced MAPKs activation via activation of the phosphatidylinositol 3-kinase pathway in murine RAW 264.7 macrophage cells. PLoS ONE.

[94] Vistica D.T., Skehan P., Scudiero D., Monks A., Pittman A., Boyd M.R. (1991). Tetrazolium-based assays for cellular viability: A critical examination of selected parameters affecting formazan production. Cancer Res..

[95] Cao Y., Chen J., Ren G., Zhang Y., Tan X., Yang L. (2019). Punicalagin prevents inflammation in LPS-induced RAW264.7 macrophages by inhibiting FoxO3a/autophagy signaling pathway. Nutrients.

[96] Burnette W.N. (1981). “Western blotting”: Electrophoretic transfer of proteins from sodium dodecyl sulfate—Polyacrylamide gels to unmodified nitrocellulose and radiographic detection with antibody and radioiodinated protein A. Anal. Biochem..

[97] Mohamed D.I., Abou-Bakr D.A., Ezzat S.F., El-Kareem H.F.A., Nahas H.H.A., Saad H.A., Mehana A.E., Saied E.M. (2021). Vitamin D3 prevents the deleterious effects of testicular torsion on testis by targeting MiRNA-145 and ADAM17: In silico and in vivo study. Pharmaceuticals.

[98] Saied E.M., El-Maradny Y.A., Osman A.A., Darwish A.M.G., Abo Nahas H.H., Niedbała G., Piekutowska M., Abdel-Rahman M.A., Balbool B.A., Abdel-Azeem A.M. (2021). A comprehensive review about the molecular structure of severe acute respiratory syndrome coronavirus 2 (SARS-CoV-2): Insights into natural products against COVID-19. Pharmaceutics.

[99] Gaber A., Refat M.S., Belal A.A.M., El-Deen I.M., Hassan N., Zakaria R., Alhomrani M., Alamri A.S., Alsanie W.F., Saied E.M. (2021). New mononuclear and binuclear Cu (II), Co (II), Ni (II), and Zn (II) thiosemicarbazone complexes with potential biological activity: Antimicrobial and molecular docking study. Molecules.

[100] Healey R.D., Saied E.M., Cong X., Karsai G., Gabellier L., Saint-Paul J., Del Nero E., Jeannot S., Drapeau M., Fontanel S. (2022). Discovery and mechanism of action of small molecule inhibitors of ceramidases**. Angew. Chem. Int. Ed..

[101] Mohamed D.I., Alaa El-Din Aly El-Waseef D., Nabih E.S., El-Kharashi O.A., Abd El-Kareem H.F., Abo Nahas H.H., Abdel-Wahab B.A., Helmy Y.A., Alshawwa S.Z., Saied E.M. (2022). Acetylsalicylic acid suppresses alcoholism-induced cognitive impairment associated with atorvastatin intake by targeting cerebral MiRNA155 and NLRP3: In vivo, and in silico study. Pharmaceutics.

[102] Mohamed D.I., Ezzat S.F., Elayat W.M., El-Kharashi O.A., El-Kareem H.F.A., Nahas H.H.A., Abdel-Wahab B.A., Alshawwa S.Z., Saleh A., Helmy Y.A. (2022). Hepatoprotective role of carvedilol against ischemic hepatitis associated with acute heart failure via targeting MiRNA-17 and mitochondrial dynamics-related proteins: An in vivo and in silico study. Pharmaceuticals.

[103] Rimon G., Sidhu R.S., Lauver D.A., Lee J.Y., Sharma N.P., Yuan C., Frieler R.A., Trievel R.C., Lucchesi B.R., Smith W.L. (2010). Coxibs interfere with the action of aspirin by binding tightly to one monomer of cyclooxygenase-1. Proc. Natl. Acad. Sci. USA.

[104] Wang J.L., Limburg D., Graneto M.J., Springer J., Hamper J.R.B., Liao S., Pawlitz J.L., Kurumbail R.G., Maziasz T., Talley J.J. (2010). The novel benzopyran class of selective cyclooxygenase-2 inhibitors. Part 2: The second clinical candidate having a shorter and favorable human half-life. Bioorg. Med. Chem. Lett..

