# Supplementary material for: Development of Novel 1,3-Disubstituted-2-Thiohydantoin Analogues with Potent Anti-Inflammatory Activity; In Vitro and In Silico Assessments
Source: Molecules. 2022 Sep 23;27(19):6271. doi: 10.3390/molecules27196271 (PMC9573447; doi:10.3390/molecules27196271)
Supplement: Supplementary file 1 [file molecules-27-06271-s001.zip › molecules-1912888-supplementary.pdf]

Supporting Information

Development of Novel 1,3-Disubstituted-2-Thiohydantoin Analogues with Potent Anti-Inflammatory Activity; *In Vitro* and *In Silico* Assessments

Salma M. Khirallah [1], Heba M. M. Ramadan [2], Ahmed Shawky [3], Safa H Qahl [4], Roua S. Baty [5], Nada Alqadri [6], Amnah Mohammed Alsuhaibani [7], Mariusz Jaremko [8], Abdul-Hamid Emwas [9], and Essa M. Saied [10,11,*]

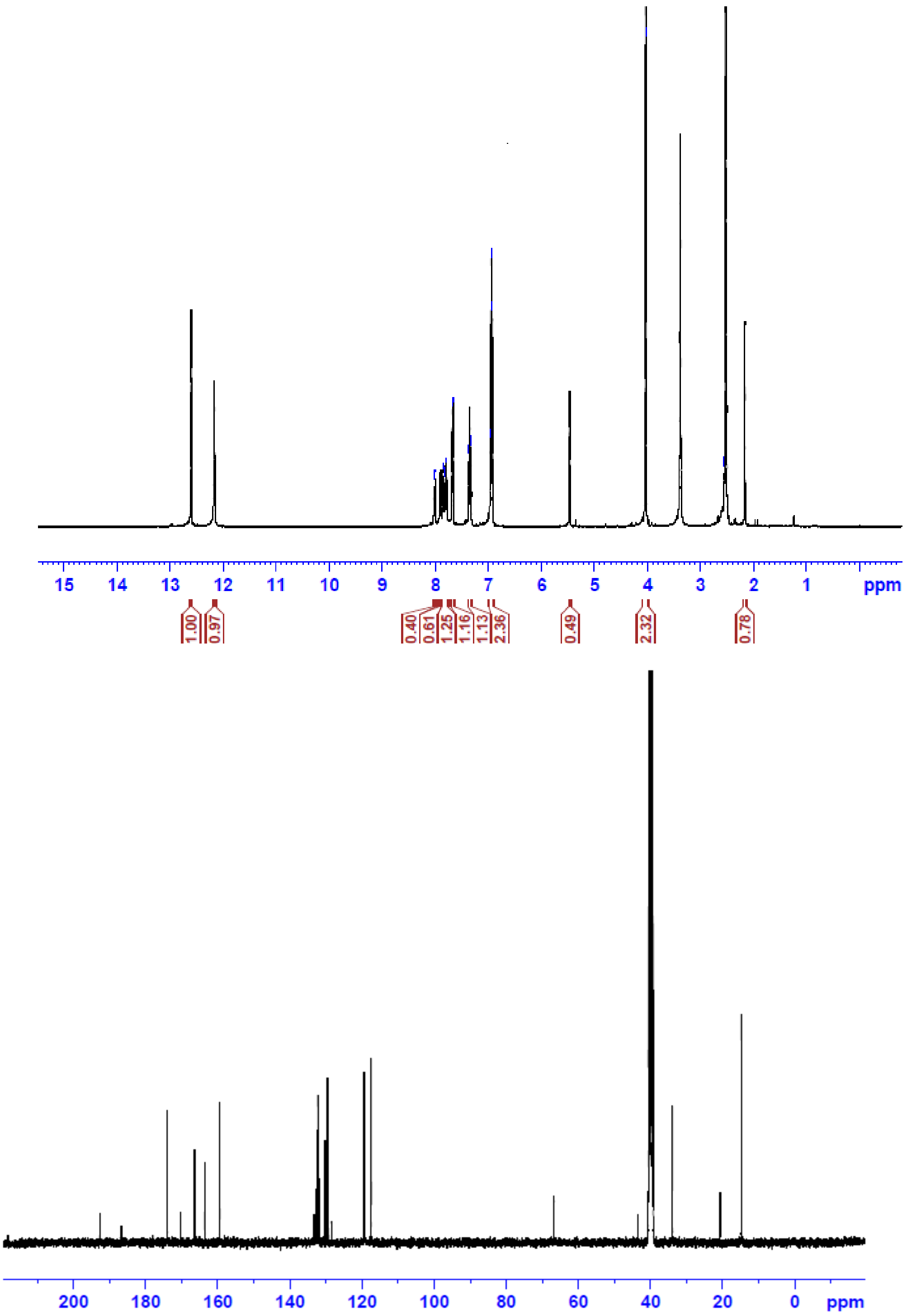

Figure S1a. **¹H-NMR and ¹³C-NMR spectra of compound 3 in DMSO-*d*₆.**

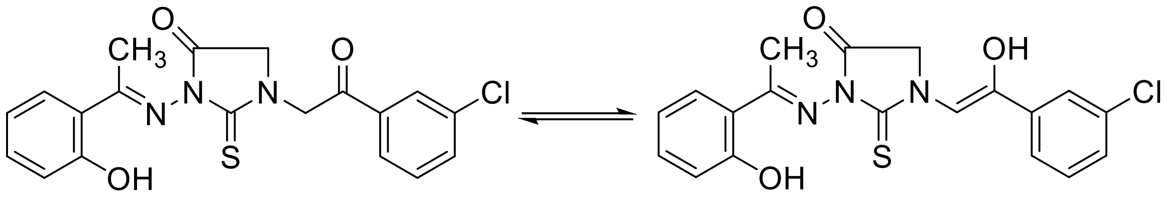

ketone form　　　　enol form

Figure S1b: The keto-enol tautomer of compound **3**.

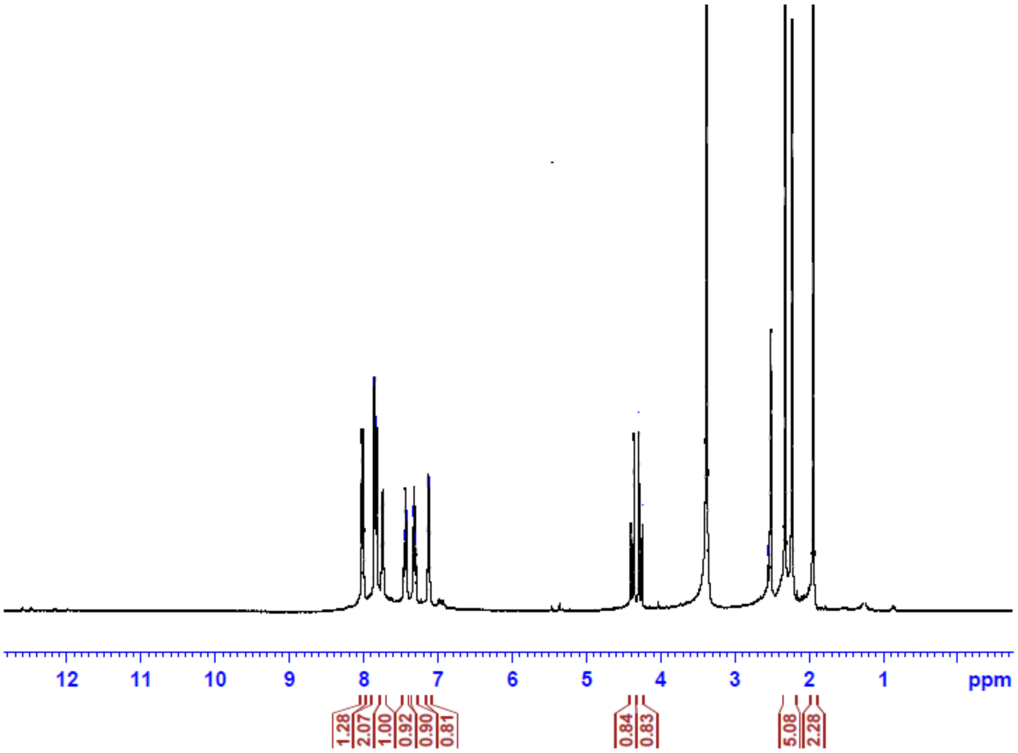

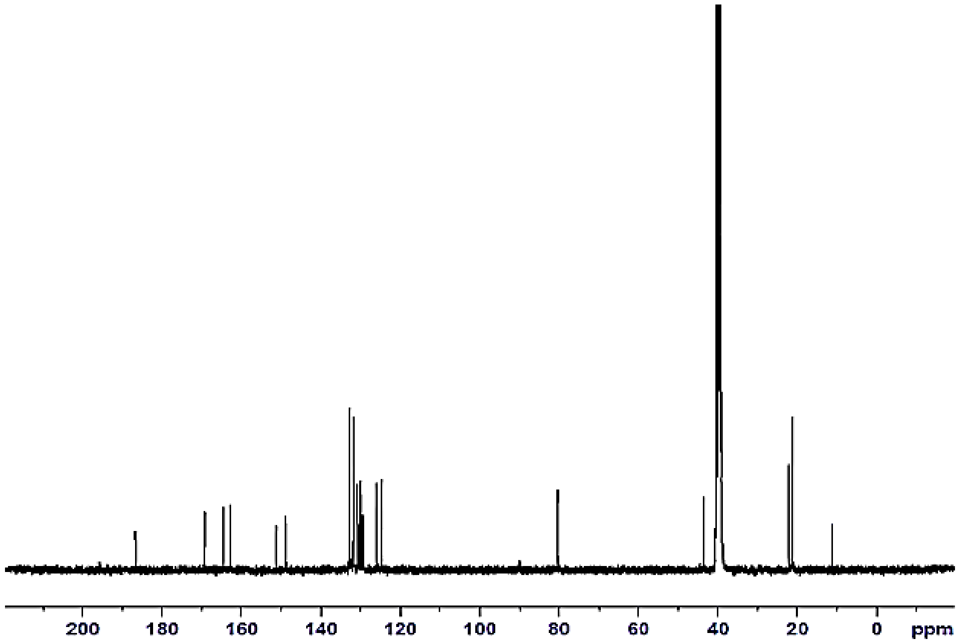

Figure S2. ^{1}H-NMR and ^{13}C-NMR spectra of compound 4 in DMSO-d_6.

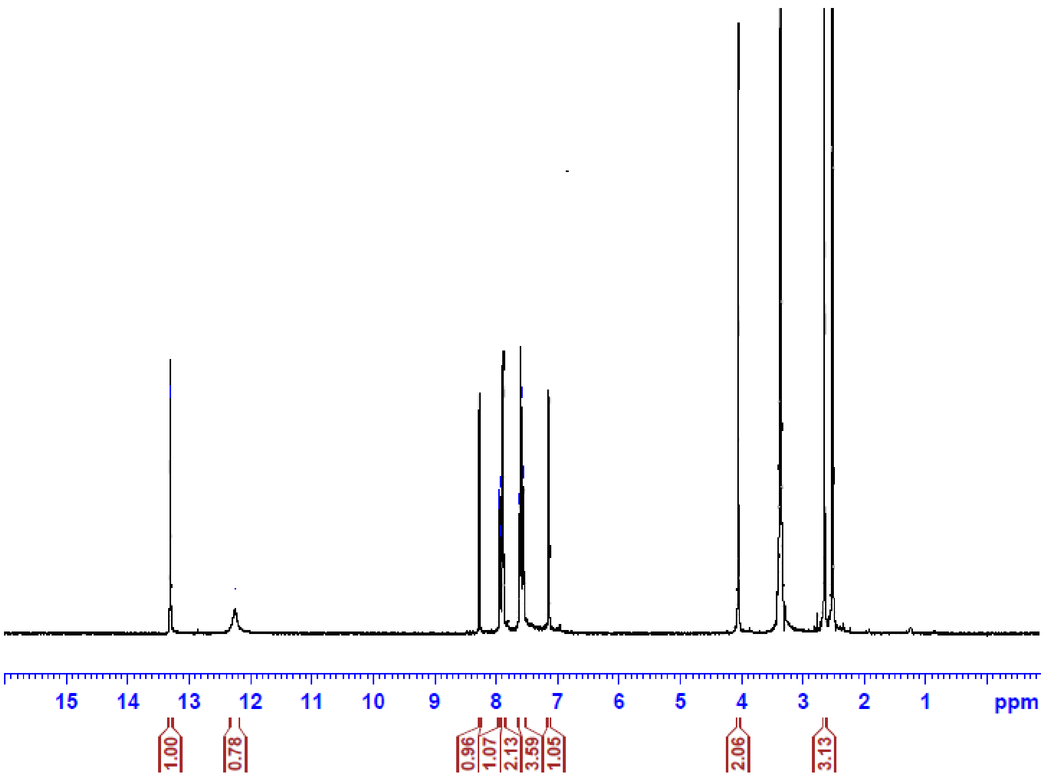

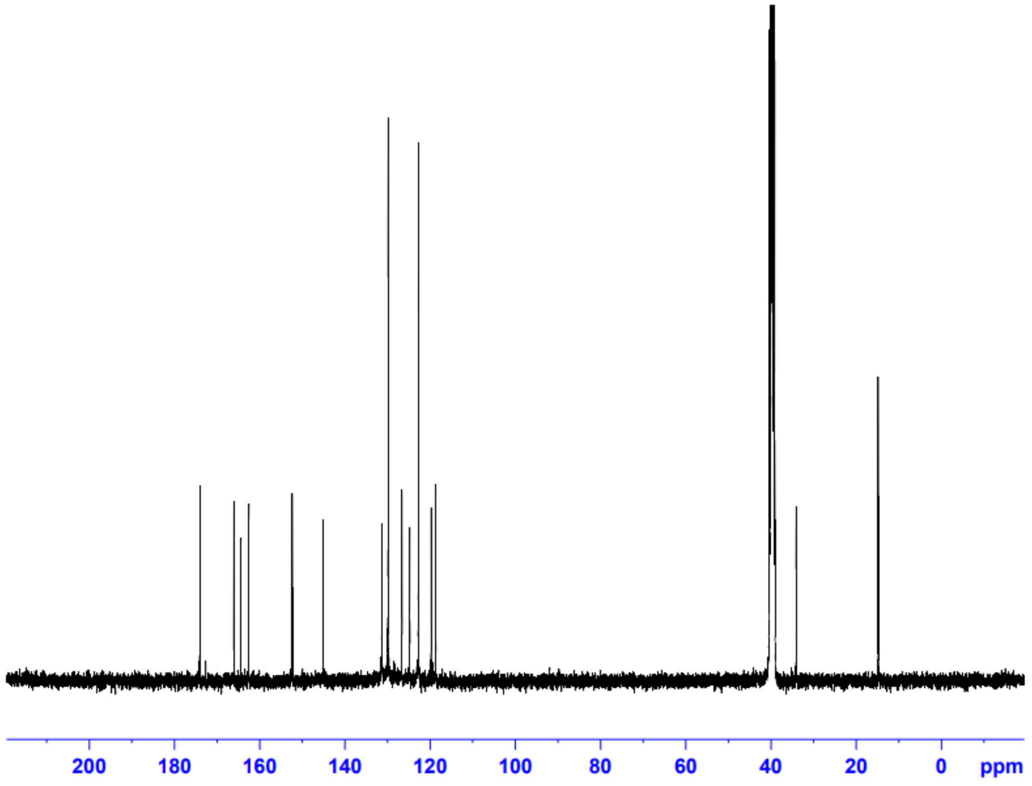

Figure S3. ^{1}H-NMR and ^{13}C-NMR spectra of compound 5 in DMSO-*d_6*.

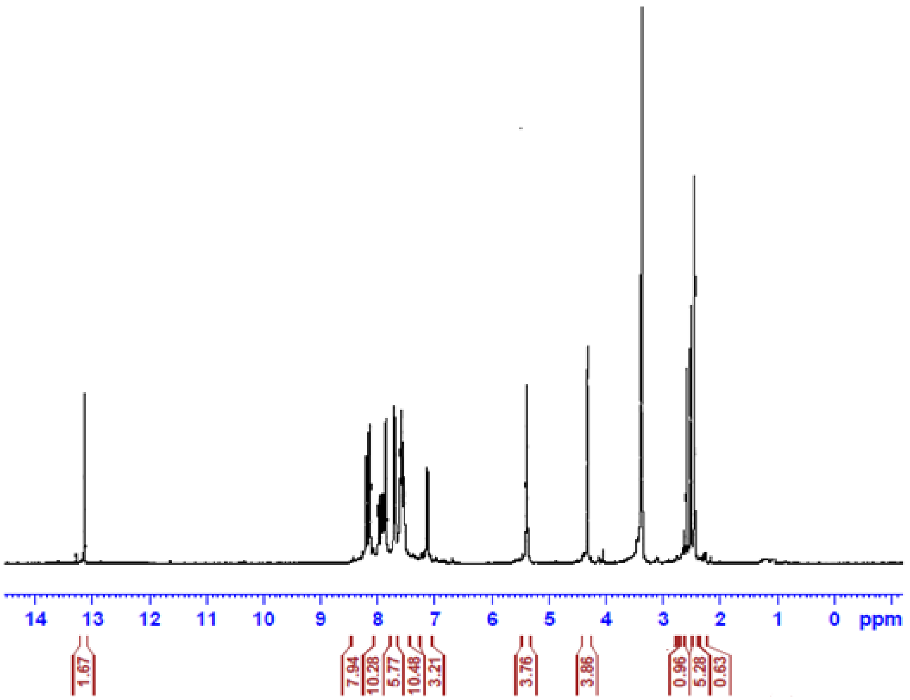

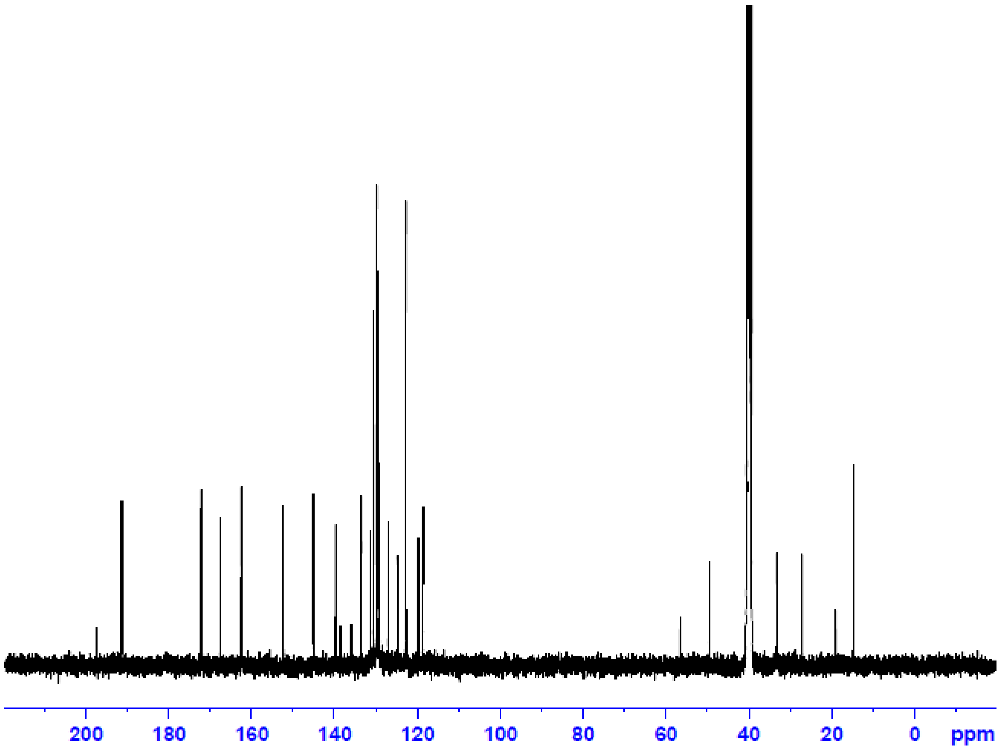

Figure S4. ^{1}H-NMR and ^{13}C-NMR spectra of compound 6 in DMSO-d_6.

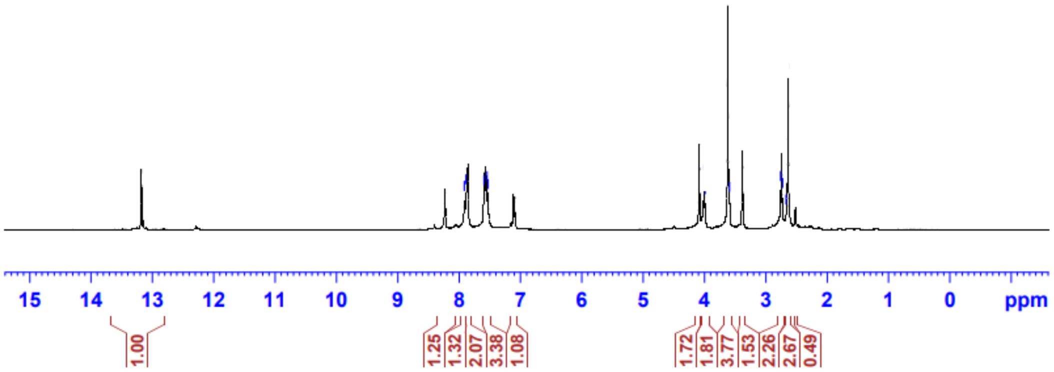

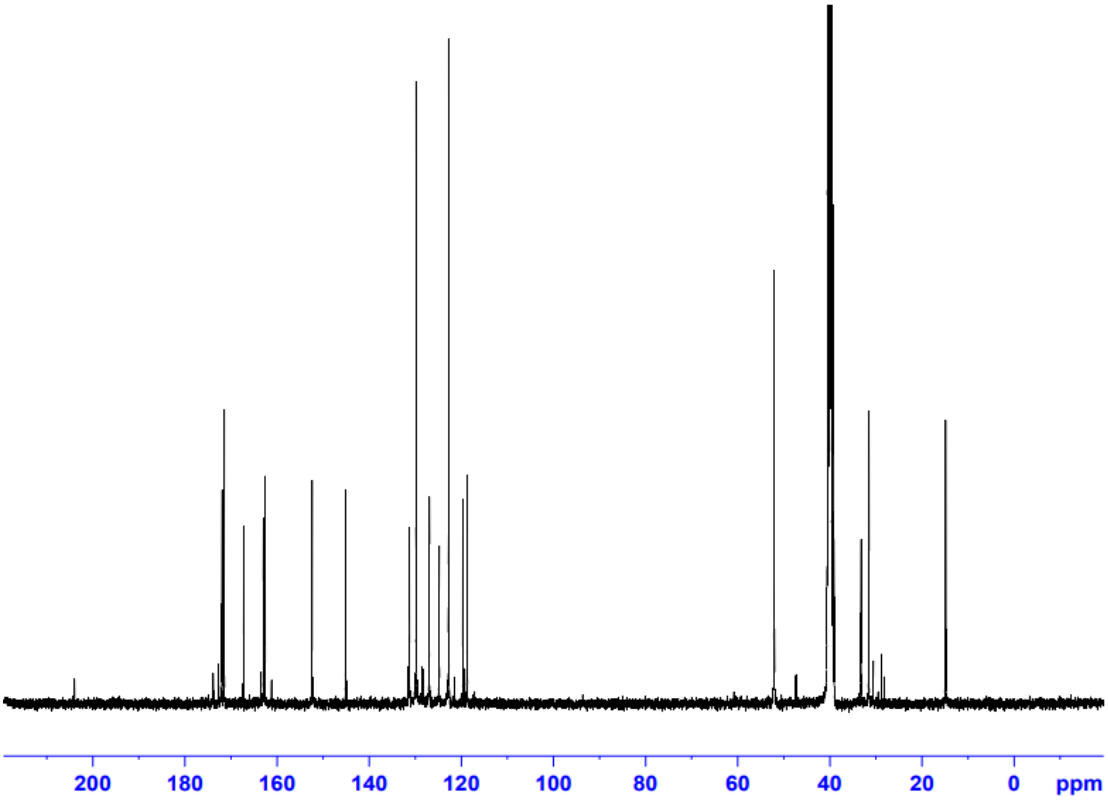

Figure S5. ^{1}H-NMR and ^{13}C-NMR spectra of compound 7 in DMSO-*d*$_6$.

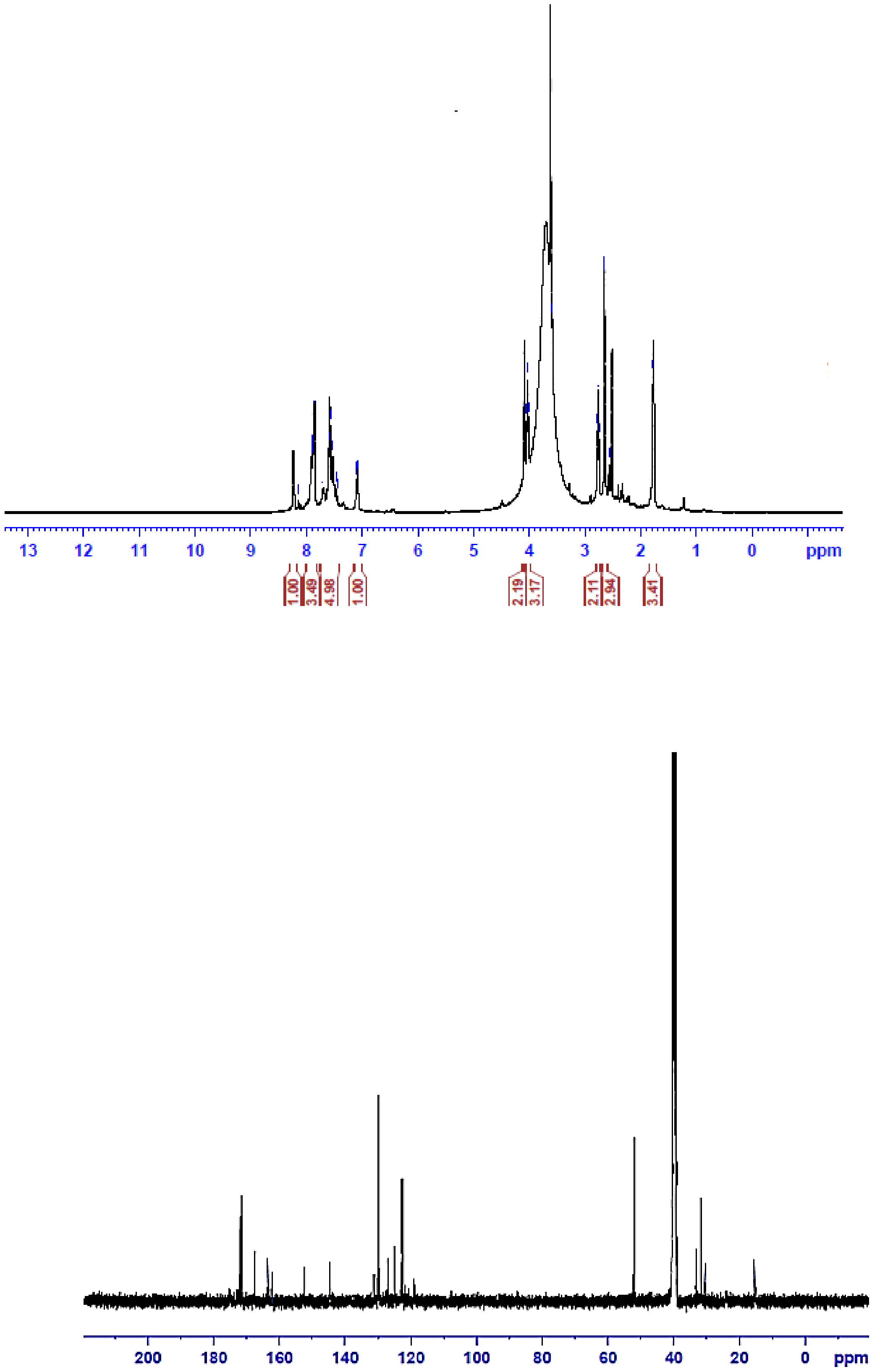

Figure S6. ^{1}**H-NMR and** ^{13}**C-NMR spectra of compound 8 in DMSO-d_6.**

Table S1: *In vitro* cytotoxicity of 1,3 disubstituted-2-thioxoimidazolidin-4-one derivatives against LPS-induced RAW264.7.

Compound 3

log conc.	% viability
3	45.09
2.398	54.99
1.799	67.86
1.204	78.37
0.6	90.78

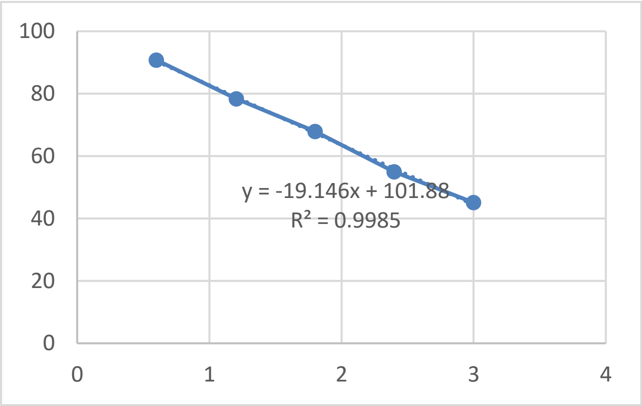

Compound 4

log conc.	% viability
3	43.27
2.39794	51.55
1.79934	63.07
1.20412	72.57
0.6	84.35

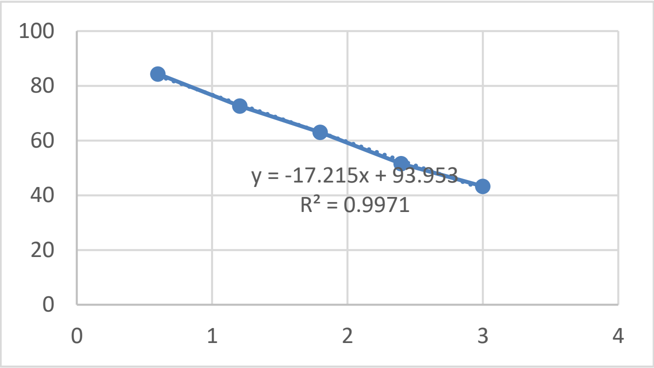

Compound 5

log conc.	% viability
3	39.26
2.398	47.99
1.799	58.86
1.204	68.82
0.6	80.66

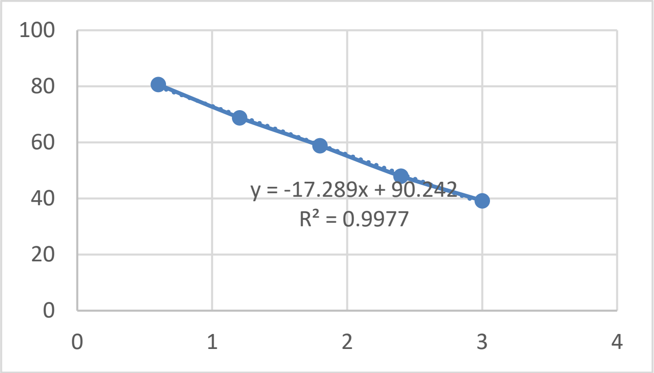

Compound 6

log conc.	% viability
3	41.6
2.39794	49.25
1.79934	61.26
1.20412	66.48
0.6	77.99

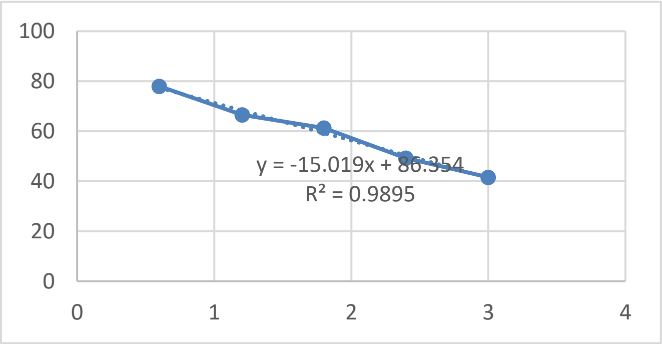

Compound 7

log conc.	% viability
3	40.06
2.39794	48.44
1.79934	55.83
1.20412	66.26
0.6	78.9

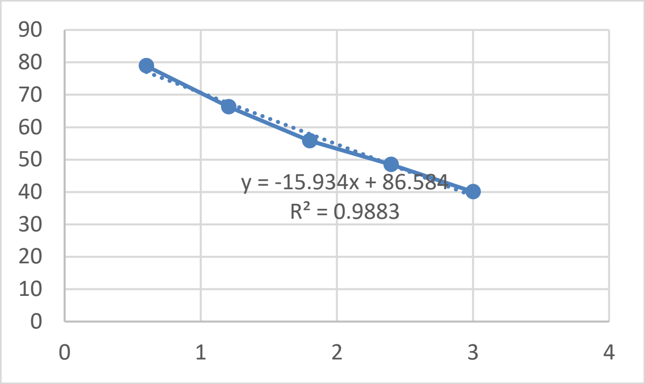

Compound 8

log conc.	% viability
3	41.54
2.398	53.67
1.799	63.56
1.204	73.13
0.6	84.83

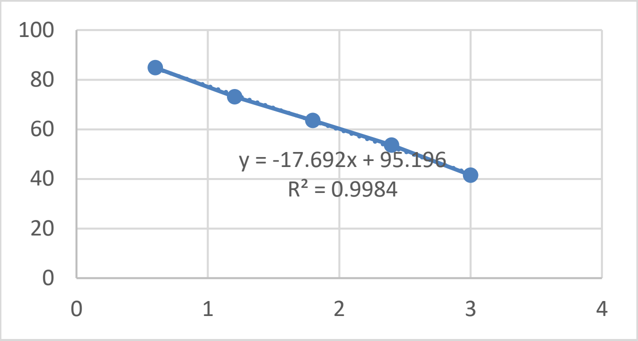

CXB

log conc.	% viability
3	41.32
2.398	48.25
1.799	59.79
1.204	70.51
0.6	79.25

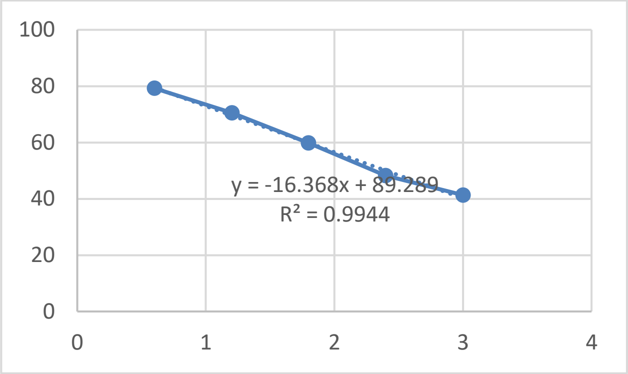

Table S2: Effect of compound 7 on IL-6 and TNF-α expression in RAW264.7 by RT-PCR.

	LPS-induced raw264.7 cells				
	Sample			Gene Expression	
				Fold of change	
		conc ug/ml	cells	IL6	TNFα
1	**7**	50		**0.276**	**0.314**
2	**Celecoxib**	50		**0.353**	**0.484**
3	**Positive Control**	---		**1**	**1**

Table S3: In vitro effect of compound 7 on Western blot analysis of IL-1β in RAW264.7 cells.

	Compound			western blotting	β-actin
	code	conc ug/ml	cells	IL1B	
				OD	
1	7	10	raw264.7	0.395	
2	7	50		0.341	+
3	PC	---		0.709	+

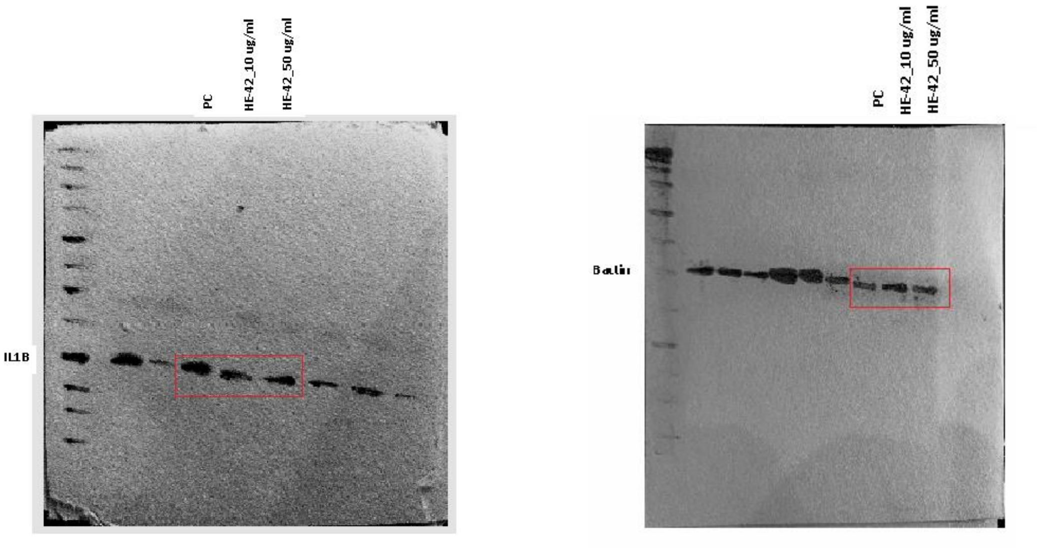

Figure S7. Western plot analysis of the *in vitro* Anti-inflammatory effect of compound ***7*** against LPS- activated RAW264.7 cell line on IL-1β expression.
